# Influences on uptake of cancer screening in mental health service users: a qualitative study

**DOI:** 10.1186/s12913-016-1505-4

**Published:** 2016-07-12

**Authors:** Abigail Clifton, Caroline Burgess, Sarah Clement, Ruth Ohlsen, Pras Ramluggun, Jackie Sturt, Paul Walters, Elizabeth A. Barley

**Affiliations:** Florence Nightingale Faculty of Nursing and Midwifery, King’s College London, 57 Waterloo Road, London, SE1 8WA UK; Division of Health and Social Care Research, Faculty of Life Sciences & Medicine, King’s College London, Addison House, Guy’s Campus, London Bridge, London, SE1 1UL UK; Institute of Psychiatry, Psychology and Neuroscience, King’s College London, Denmark Hill, London, SE5 8AF UK; Faculty of Health and Life Sciences, Oxford Brookes University, Jackstraws Lane, Marston, Oxford, Oxon OX3 0FL UK; Bournemouth University and Dorset HealthCare University NHS Foundation Trust, Sentinel House, Nuffield Road, Poole, BH17 0RB UK; College of Nursing, Midwifery and Healthcare, University of West London, Paragon House, Boston Manor Road, Brentford, Middlesex, TW8 9GA UK

**Keywords:** Mental health, Preventative medicine, Public health, Qualitative research, Cancer screening, Breast, Cervical, Bowel

## Abstract

**Background:**

Cancers are a leading cause of death worldwide. People with mental illness are 30 % more likely to die from cancer than the general population. One reason for this may be low uptake of nationally offered cancer screening tests by people with mental illness. We aimed to identify barriers and facilitators for breast, cervical and bowel cancer screening uptake by people with mental illness in order to inform interventions to promote equal access.

**Methods:**

The interview study was conducted in both urban and rural settings. The study was informed by the Theoretical Domains Framework, using framework analysis and triangulation across participant groups. Participants included 45 mental health service users (service users) eligible for cancer screening, 29 mental health professionals and 11 professionals involved in cancer screening.

**Results:**

Themes emerging from the data that affected uptake included knowledge of screening programmes by both service users and healthcare providers; knowledge of, and attitudes towards, mental illness; health service-delivery factors; service users’ beliefs and concerns about cancer screening, and practical issues. These are relevant to different stages of the screening process. Service users do not receive invitations to screening or cancer testing kits if they are admitted to hospital. They are not routinely invited for screening if they are not registered with a general practitioner (GP). Lack of integrated care means that mental health staff do not know if someone is overdue for a test and cancer screening is often not considered during health promotion. Barriers including information processing problems, the extent to which the screening process aggravates symptoms, poor staff client relationships and travel difficulties vary between individuals. Screening professionals are motivated to help, but may lack time or training to manage mental health needs. Reactive measures are available, but service users must request help which they may find difficult.

**Conclusions:**

There are specific barriers to cancer screening uptake for mental health service users that prevent equality of care. Interventions that can be personalised are needed at individual, policy and service-delivery levels. Primary and secondary care staff and policy-makers should work together to develop an integrated approach to cancer screening in this population.

**Electronic supplementary material:**

The online version of this article (doi:10.1186/s12913-016-1505-4) contains supplementary material, which is available to authorized users.

## Background

Cancers are a leading cause of morbidity and mortality worldwide [1]. There is conflicting evidence regarding the incidence of cancer in people with mental illness with studies finding the risk of cancer to be higher, lower, or equivalent to that of the general population [2]. However, it has been found that people with a mental illness diagnosis who are in contact with mental health services are 30 % more likely to die from cancer than the general population [3]. Lifestyle factors such as smoking, obesity, alcohol use, poor diet and lack of exercise in service users are likely to increase morbidity and mortality [3–10], however it is likely that there are other contributing factors [3], such as low uptake by service users of cancer screening tests offered nationally [11, 12] leading to later presentation. Results from recent data syntheses and research articles indicate that inequalities exist in breast and cervical cancer screening uptake in women with a range of different mental health diagnoses in comparison to the general population. Xiong et al. (2008) also considered prostate and colorectal (bowel) cancer screening (including faecal occult blood test) in people with mental illness finding that uptake was particularly low for bowel cancer screening [13]. Poor engagement with treatment could also be associated with this increased mortality; depression has been found to be a risk factor in poor engagement with medical treatment [14] however this is beyond the scope of the current study.

Systematic reviews [15–17] have demonstrated the effectiveness of a range of interventions to increase cancer screening uptake in the general population. However, a Cochrane review [18] conducted by two of the current authors (EB, PW) found no trials of interventions to increase cancer screening uptake in mental health service users. As a first step towards developing such an intervention, it is necessary to identify barriers and facilitators to cancer screening uptake for people with a mental illness diagnosis.

Several studies have considered reasons for cancer screening non-uptake in people with mental illness. These have concluded that certain reasons such as low income, lack of transport and embarrassment may be shared with other disadvantaged groups [19–22]. However, others suggest that there are population-specific reasons that could be addressed. For instance, studies conducted in the US [23, 24] suggest that health service delivery factors, such as poor communication between primary care and psychiatric services, are important. A survey of mental health service and cancer screening providers in London, which focused on people with mental illness within the British capital’s African Caribbean communities [25], suggests that this may also be the case in the UK. However, no UK study has considered this from a mental health service user perspective.

Other barriers to health care that are specific to this population include stigma and discrimination from healthcare professionals and difficulty seeking, comprehending and acting upon advice as a result of symptoms and cognitive deficits associated with a range of mental health diagnoses [5]. It is not known whether this applies to cancer screening uptake. Furthermore, the few published studies that focus on cancer screening in people with mental illness [11, 12] have mostly considered breast and cervical cancer screening and not bowel cancer screening, which is a national programme in several countries and may present specific challenges.

Equality of access is policy for national screening programmes. If we are to ensure this, clinicians, service providers, policy-makers and researchers need to understand which factors help or hinder uptake. This study was conducted in order to identify the barriers and facilitators perceived and experienced by people with a diagnosis of mental illness to their uptake of a range of cancer screening tests.

## Methods

### Design

Qualitative interview study of people with mental illness eligible for cancer screening, mental health professionals and professionals involved in the screening process. Potential ethical dilemmas were taken into consideration in the design and conduct of this study. The study was approved by the London Bridge Research Ethics Committee (REC number 14/LO/1511) on October 1st 2014. Research and Development (R&D) approval was provided by King’s College Hospital, Dorset University Hospital Foundation and South London and the Maudsley NHS Trusts. All participants had at least 24 h to read the participant information sheet and choose whether they would like to take part. All participants provided written informed consent. Where exemplar quotes are provided participants provided written consent for these to be used in reports of the results of the study.

### Public Involvement

One author is a mental health service user. A further mental health service user was recruited to our project review group. Both have advised on rationale, methods, materials, dissemination of findings and the project review group member helped identify potential participants.

### Sampling and Recruitment

#### Mental health service users (service users)

The sampling frame comprised people with mental illness with a degree of severity resulting in accessing secondary care (such as a Community Mental Health Team (CMHT)) in the previous five years. Given the uncertainty around mental illness classifications, the frequency of co-morbidity, and because future service changes or interventions are likely to be targeted across populations we included individuals with a range of diagnoses. Eligible self-reported diagnoses were bipolar disorder, schizophrenia, other psychoses, borderline personality disorder, major depression and severe anxiety disorders. Participants also needed to be eligible for cancer screening via three national cancer screening programmes (breast, cervical and bowel). The cervical cancer screening programme is offered to women aged 25–65 years. Mammography is offered to women aged 50–70 years. Bowel cancer screening using the faecal occult blood test is offered to men and women aged 60–74 years. We purposively sampled for age and gender in both rural and urban settings in the service user sample, and for location and profession in the professional samples. Participants were recruited via posters in CMHT waiting rooms and inpatient wards within two NHS mental health trusts (one in London and one in a rural, costal area of Dorset). Letters were also sent to CMHT service users who had taken part in an earlier London based study [26] and who had consented to be informed about future studies. A snowball sampling method [27], whereby existing study participants recruit future participants from among their acquaintances, was also implemented.

#### Professionals

The sampling frame included professionals working in the two aforementioned NHS trusts and who are involved in undertaking, promoting, or potentially promoting cancer screening. Screening professionals, e.g. GPs, practice nurses, sexual health nurses and breast screening unit staff, and mental health professionals of any discipline who were involved in promoting physical health reviews were recruited via posters and flyers left in clinical areas, and via snowballing.

### Data collection

Three qualitatively trained female researchers (AC, Dorset based Research Nurse and Research Assistant) conducted in-depth interviews, either face to face or over the telephone informed by an interview schedule (Additional file 1a, b, c). Participants were given the option of being interviewed by a male if they wished. Interviewers were aged 25, 50 and 53; all were white British. Interviewers were trained together and interviewing techniques were practiced, though it is recognised that it is unavoidable that interviewer characteristics may have influenced the data collected. Interviews were audio recorded and transcribed verbatim, with field notes recorded post interview. Data collection and analysis were iterative and continued until theoretical saturation (no novel findings) had been reached.

### Interview Schedule

The Theoretical Domains Framework (TDF) [28] informed the interview schedules. This covers a set of domains comprising evidence-based factors influencing behaviour change, such as knowledge, beliefs about the consequences of the target behaviour, social influences such as the attitudes of close others, and the environmental context. The framework allows researchers to explore the content of each domain with respect to the particular behaviour of interest, in this case the decision whether to attend for cancer screening. Use of this evidence-based tool was designed to ensure that any future intervention to promote cancer screening uptake informed by this study would be theory-based.

### Data analysis

Framework analysis [29] was conducted. After familiarisation with the interview data, an analytic framework, broadly based on the domains that generated the interview schedule, was used by AC to organise the data according to key categories and a set of codes was produced and agreed within the multidisciplinary team which included a service user researcher (AC, EB, SC, CB, JS). The codes were systematically applied to the dataset (indexing). Data was then summarised into a “case by category” matrix on a spreadsheet (charting) for interpretation and the development of explanatory themes. Each transcript was coded by one researcher (AC) and a sample of 30 % was independently assessed by another researcher (EB, PR, RO) to ensure agreement. Themes and associated barriers and facilitators were identified from reading the summaries in the charts and discussion within the multidisciplinary research team. We actively sought evidence of disconfirming data and sought out examples of barriers and facilitators that appeared specific to either screening type or participant characteristics. Relevant participant quotes to illustrate themes were agreed by the team.

This process was conducted for each sample. We then used a triangulation approach combining barriers and facilitators from patient and professional interviews to identify overarching themes. This involved producing a ‘convergence coding matrix’ to display barrier and facilitator findings from the different samples together. Finally, we considered where there was agreement, partial agreement, silence (a finding in one sample only) or dissonance between the samples.

## Results

Eighty-five people were interviewed: 45 service users (29 in London), 11 screening professionals (8 London, 2 Kent and 1 Oxfordshire), 29 mental health professionals (19 London). Interviews were conducted face to face (30 service users, 10 screening professionals, 20 mental health professionals) or over the telephone (15 service users, 1 screening professional, and 9 mental health professionals).

### Participant Characteristics

The characteristics of the service users are shown in Table 1.

**Table 1:** Characteristics of service user participants

	London (*n* = 29) n/range (mean)	Dorset (*n* = 16) n/range (mean)	Total (*n* = 45) n (%)/range (mean)
Gender			
Female	26	13	39 (87 %)
Male	3	3	6 (13 %)
Age	33–70 (51)	26–73 (46)	26–73 (49)
Ethnicity^a^			
White British	15	16	31 (69 %)
Black or Black British – African	3	0	3 (7 %)
Black or Black British – Caribbean	5	0	5 (11)
Mixed – white and Black African	2	0	2 (4 %)
Mixed – white and Black Caribbean	2	0	2 (4 %)
Other^b^	2	0	2 (4 %)
Diagnosis^c^			
Schizophrenia	2	1	3 (7 %)
Schizoaffective disorder	4	1	5 (11 %)
Bipolar disorder^d^	12	3	15 (33 %)
Other psychosis	1	1	2 (4 %)
Depression^e^	4	3	7 (16 %)
Depression & anxiety	3	2	5 (11 %)
Anxiety disorder	1	1	2 (4 %)
Personality disorder^d^	2	1	3 (7 %)
Personality disorder & depression^e^	0	3	3 (7 %)
Duration of diagnosis (years)	1–50 (20)	6–44 (15)	1–50 (19)
Current mental healthcare			
Inpatient	4	10	14 (31 %)
Community Mental Health Team	10	6	16 (36 %)
Primary care	15	0	15 (33 %)
Type(s) of screening discussed			
Cervical only	16	9	25 (56 %)
Breast only	3	0	3 (7 %)
Bowel only	3	3	6 (13 %)
Cervical and breast	7	3	10 (22 %)
Cervical, breast and bowel	0	1	1 (2 %)

^a^Self-reported

^b^Arab (*n* = 1), Asian or black British – Indian (*n* = 1)

^c^Self-reported, apart from in one case where participant did not know and information was obtained from clinical staff with participant consent

^d^Including 1 with co-morbid ADHD

^e^Including 1 with co-morbid anorexia

A total of 45 service user participants, 39 female (87 %) (*n* = 26 London, 13 Dorset), six male (*n* = 3 London, 3 Dorset) were interviewed. Ages ranged from 26–73 years with a mean age of 49 years. In the London sample the age range was 33–70 years with a mean age of 51 years. In the Dorset sample the age range was 26–73 years with a mean age of 46 years. More women than men were eligible for this study due to the sampling frames used. A total of 36 participants discussed cervical cancer screening; this is the largest group due to having the widest sampling frame (ages 25–65); a total of 14* participants discussed breast screening (sampling frame was ages 50–70); and a total of seven* participants bowel screening. This is the smallest group and has the most limited sampling frame (ages 60–74). Of these, 11 participants elected to discuss more than one screening type.

One service user (from Dorset) was not registered with a GP. Twelve service users reported a family history of cancer. Our sample includes service users who reported having missed, declined, ignored, or delayed cancer screening as well as those who had received screening on time.

The 11 screening professionals (10 female) were aged between 31 and 67 (mean 48) years, with between 10 and 30 (mean 19) years’ experience. Most worked in London, but we also recruited via snowballing, two from Kent and one from Oxfordshire. Professions included GP (*n* = 1), practice nurse (*n* = 2), sexual health clinic nurses (*n* = 2), radiographers (*n* = 3), breast screening service managers (*n* = 2) and a senior public health employee (*n* = 1).

The 29 mental health professionals (19 female) were aged between 26 and 56 years (mean 42 years), with between 0.5 and 30 (mean 11) years’ experience. Nineteen worked in London. Staff recruited were: mental health nurse/practitioner (*n* = 11), social worker (*n* = 1), occupational therapist (*n* = 3), psychiatrist (*n* = 1), support worker/nursing assistant (*n* = 6), inpatient unit housekeeper (*n* = 1), therapeutic benefits coordinator (*n* = 1), mental health nursing researcher (*n* = 3) and student nurse (*n* = 2).

### Themes

**Fig. 1:**
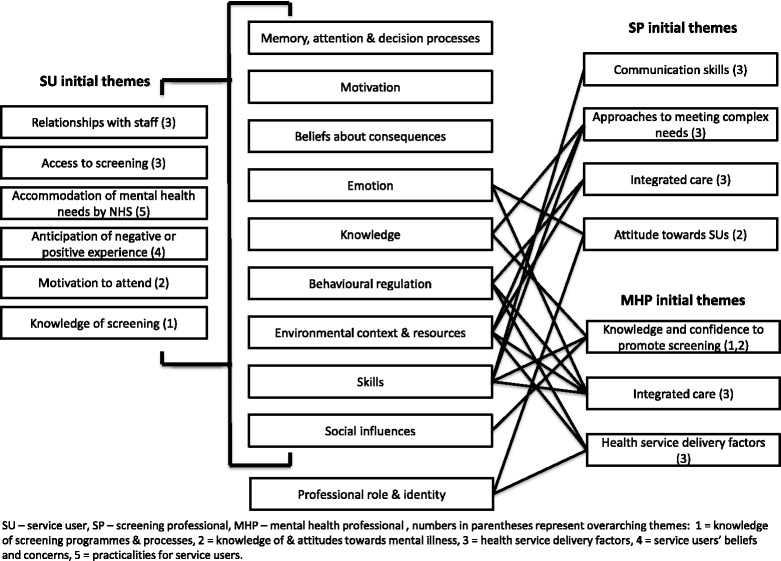
Initial themes by participant group and their relationship to the Theoretical Domains Framework [28]

Overall themes appeared to be independent of any participant characteristic, including diagnosis. For example, specific mental health symptoms sometimes affected participants’ attendance of appointments in different ways but the barrier remained the same. We therefore identified themes for the service user population as a whole. Initial themes, their associated barriers and facilitators and their relationship to the Theoretical Domains Framework are shown for each sample in Fig. 1 and in detail in Additional file 2: Tables S1 to S3. All theoretical domains were represented in the raw data for the service user and professionals groups. For the key findings the following theoretical domains were represented in the service user data: knowledge; skills; social influences; memory, attention and decision processes; beliefs about consequences; motivation; emotion; behavioural regulation and environmental context and resources. The professional data differed in that memory, attention and decision processes; beliefs about consequences and motivation were not represented and professional role and identity were represented. The theoretical domains not represented in our key findings were beliefs about capabilities and nature of the behaviour [28]. Themes commonly related to more than one type of cancer screening, differences between screening types are mainly related to the method of screening delivery. For example, fewer barriers regarding making appointments and interacting with professionals were found for the bowel cancer screening group as this test is completed at home. Similar barriers and facilitators arose from the data gathered from participants in Dorset and London; identified differences are reported in the text.

Triangulation of the barriers and facilitators resulted in identification of five overarching themes. Overarching themes and constituent barriers and facilitators are shown in Table 2 (the links to the initial themes within the participant groups are shown in Fig. 1). There was at least partial agreement between the samples for each theme, but disconfirming evidence is reported. We provide an overview of these themes supported by illustrative quotes which are identified by participant number and gender (M = male/F = female). For professionals participant number, role and location (L = London or D = Dorset) is stated. Further exemplar quotations are available in Additional file 3.

**Table 2:** Overarching themes and constituent barriers and facilitators to cancer screening uptake

Theme: Knowledge of screening programmes and processes	
Constituent barriers	Constituent facilitators
SU: Not knowing what to expect or what to do; unsure of need for screening; difficult to process information SP: Communication skills training not available to all MHP: Lack of knowledge of programme and/or procedures; promotion of screening not prioritised; lack of physical health expertise	SU: Wanting to be informed; understanding the benefits of screening; feeling health conscious; encouragement MHP: Health promotion seen as their role; aware that SU are at risk of cancer; understanding emotional and practical barriers to screening uptake for SU
Theme: Knowledge of, and attitudes towards mental illness	
Constituent barriers	Constituent facilitators
SU: Lack of understanding of mental illness in screening professionals; made to feel like a burden on health service; stigma of mental illness SP: Lack of knowledge of severe mental illness; find complex patients difficult MHP: Stigma of mental illness (among others)	SU: Staff being understanding; staff knowledge of mental illness SP: Understanding of emotional and practical barriers to screening uptake for SU; staff motivated to encourage screening for all groups; importance of good communication skills recognised; confidence to screen anyone associated with good communication skills
Theme: Health service delivery factors	
Constituent barriers	Constituent facilitators
SU: Screening environment aggravates mental health symptoms; staff can be rushed; staff can be rough; exclusion from GP registers SP: Lack of time; no means of knowing patient needs in advance; computer systems not linked MHP: Lack of a structured behaviour change approach; lack of collaboration between healthcare services; no one has clear responsibility to promote screening; patient’s mental state; lack of resources	SU: Continuity of care SP: Practice nurses can access patients’ records; reactive measures in place if advance notice of need is given MHP: Diagnostic overshadowing known to be a problem; willingness to promote screening; cancer screening promotion included in routine health promotion
Theme: Service users’ beliefs and concerns	
Constituent barriers	Constituent facilitators
SU: Additional burden; mental health symptoms reduce motivation for self-care; past negative experience; embarrassment; traumatising; fear of bad news; poor relationship with GP; diagnostic overshadowing	SU: Feeling health conscious; being anxious to avoid further health problems; physical symptoms (e.g. finding a lump); past positive experience; good relationship with GP; good relationship with practice nurse SP/MHP: Awareness of some of these difficulties
Theme: Practicalities for service users	
Constituent barriers	Constituent facilitators
SU: Appointment booking; transport difficulties; difficulty remembering appointments; difficulty leaving the house due to mental health problems; taking time off	SU: Familiar location; reminders SP/MHP: Awareness of some of these difficulties

*SU* service user, *SP* screening professional, *MHP* mental health professional

### Knowledge of screening programmes and processes

Some service users reported not knowing what to expect or do with regard to attending screening: “*I thought … they were going to put me through a tunnel*” (*P4, F, L*). Others were unsure of the need for screening, and some had difficulty understanding information due to poor concentration. A lack of knowledge of the screening programme was highlighted by most mental health professionals: “*to be honest I don’t know much at all*” (*P50, Psychiatrist*, *L*). Some noted they lacked physical health expertise in general: “*mental health nurses’ confidence in addressing physical health issues is probably quite low*” (*P18, Mental Health Nurse, L*).

In contrast, some service users understood the benefits of screening, which were identified as living longer, being healthier or avoiding further illness. Encouragement from friends, family or healthcare professionals was said to facilitate screening attendance: “*it’s my friend and my mum who will push me and say you should get this sorted out*” (*P307, F, D*). Mental health professionals agreed that health promotion was their role and demonstrated awareness that service users are at risk of cancer.

### Knowledge of, and attitudes towards, mental illness

Service users felt screening staff often lacked understanding of their needs, symptoms or the side effects that they experience: “*the lady was laughing because I was shaking …I told her I was on medication that causes my hands to tremor. She could have been more understanding*” (*P3, F, D*). It was reported that healthcare staff in general could make them feel like they were a burden on the health service: “*I feel that I’m taking up his [GP] time…*” (*P24, F, L*). Mental health professionals also raised this issue noting that non-mental health care professionals often have stigmatising attitudes towards service users: “*There’s the general attitude towards mental illness in primary care… for example, forensic histories and mental, quite severe and enduring anti-social histories are best avoided*” (*P17, Mental Health Nurse, L*). Some screening professionals also said they lacked training in mental health and that they found meeting the needs of complex patients difficult: “*… they seem very needy and they need to sit down and can I have some more time and it’s just a mammogram obviously to me*” (*P56, Mammographer, L*).

However, some service users reported experiences where screening staff had been understanding and when this had occurred they were motivated to return. Screening professionals were motivated to promote screening uptake among all groups and they recognised the importance of good communication skills to do this. Participants who felt that they had good communication skills were confident to screen service users: “*I feel quite confident that I’ve met a lot of different people, different personalities and it helps you to adapt in different situations*” (*P58, Mammographer, L*). Communication skills training is available in some services, but not necessarily for all staff.

### Health service delivery factors

Service users were concerned about how screening tests were sometimes delivered; there were reports that staff can be rushed and the procedure rough: “*I’ve never had such a rushed appointment in my life compared to the mammogram.... it’s really left me with quite significant trauma*” (*P35, F, L*). Noisy screening environments can aggravate mental health symptoms: “*I may have paranoia, so waiting in a room full of other people … that’s not good…*” (*P22, F, L*) and “*I’m a voice hearer, there are times when you can talk out to the voices so it would be difficult*” (*P23, F, L*).

The problem of being excluded from GP registers was also raised. In the UK, GPs may exclude a patient (i.e. refuse to keep them listed at the practice) if the GP decides that trust has broken down between them, for instance following multiple non-attendance at appointments made by the patient or following patient behaviour perceived to be abusive. This was only raised by participants based in London however. Exclusion led to people missing out on screening invitations if they did not register with another practice. Exclusions typically followed an incident in which the participant had been aggressive towards primary care staff. The participants felt that they weren’t always able to control themselves due to their illness and medication. Mental health professionals also discussed this: “*I had quite a few clients who got struck off by the GP because either they are perceived as not following the rules or they are perceived as rude to the receptionists*” (*P8, Honorary Consultant Nurse & academic, L*).

Mental health and screening professionals reported a lack of integration and communication between care systems. Separate computer systems were a problem for screening professionals who had no way of knowing if a patient may have specific needs. Similarly, mental health professionals do not always know whether a patient is due for screening: “*… sometimes knowing when all our patients have appointments can be a bit difficult for us unless we get a letter or they come with a letter*” (*P103, Mental Health Nurse, D*). Practice nurses are able to access patient records and find this helpful in planning care. The open ended appointments system in sexual health clinics was also seen as helpful when complex needs had to be met: “*we could see someone for 45 minutes if that person needed that time*” (*P2, Sexual Health Nurse, L*). In breast cancer screening units, reactive measures can be put in place if notice is given, for instance longer appointments and adjustments to staffing can be arranged. However, this puts the onus on the service users to notify services of their needs, which some may find difficult.

Mental health professionals reported that no one has overall responsibility for promoting cancer screening. Cancer screening is not prioritised even when engaging in health promotion: “*Our priority is, you know, engagement, stabilisation and not cancer screening*” (*P17, Mental Health Nurse, L*) and “*..we’ve been.. wrapped up with the metabolic syndrome and looking at heart disease and diabetes and not all that side of it (cancer screening) really*” (*P308, Mental Health Nurse Practitioner, D*). Nevertheless, except where service users were considered too acutely ill, a willingness among mental health staff to promote cancer screening was noted. Opportunities to do this when carrying out cardiovascular screening were acknowledged: “*I suppose it should be included in their care plan or when they have their physical health assessment*” (*P52, Student Mental Health Nurse, L*).

### Service users’ beliefs and concerns

Motivation to attend cancer screening was reduced among service users when attendance was perceived as an additional burden in their busy and stressful lives: “*It can be daunting especially when you are feeling so low..and you get these letters. Sometimes I feel my head is going to explode*” (*P15, F, L*). Several participants stated that when they feel depressed or mentally unwell they struggle to care about themselves: “*Sometimes when you are feeling low you don’t tend to look after yourself”* (*P15, F, L*). For some this was to the extent that they did not value their own lives: “*It just seems like nothing is really worth it anyway....it doesn’t matter if you were to have it (cancer) because it would do everyone a favour*” (*P23, F, L*).

Anticipation of a negative experience, for instance, one that would be traumatising, embarrassing or that would result in ‘bad news’ was a barrier; for some, these concerns were grounded in past negative experiences of cancer screening. Similarly, service users who felt that their relationship with health professionals was poor were less inclined to attend cancer screening. Poor relationships were often rooted in perceptions that staff did not take service users seriously and that diagnostic overshadowing (where professionals are too focused on mental health problems to take concerns about their physical health seriously) was a problem: “*As soon as the doctor found out that I had mental health problems he said, ‘oh all the symptoms you are experiencing are anxiety’..He refused to see me and then a week later I had to go to A&E (accident and emergency) because I’d actually got a water infection*” (*P306, F, D*). Professionals, especially mental health professionals, appeared to be aware of at least some of these concerns and reported empathy with service users.

Some beliefs and concerns were identified by service users as motivating factors to attend screening, for instance, ‘being health conscious’ and wanting to avoid further health problems: “*I don’t want added problems, you know”* (*P10*, *F, L)*; and physical symptoms such as ‘finding a lump’: *“It was only when I felt this big lump that I thought oh shit I’d better do something about it” (P307, F, D).* Positive experience of screening and good relationships with GPs or practice nurses was reported as a facilitator for cancer screening uptake: *“they also explained every single thing that they were going to do and I think that makes you automatically relax” (P309, F, D).*

### Practicalities for service users

A range of practical difficulties in attending screening were highlighted such as booking and remembering appointments: *“I’ve learnt over the years that depression messes with your memory …” (P44, F, L);* leaving the house due to mental health problems: *“.. I’ve been very low to the point whereby I don’t even want to go out the house and I’m regularly missing appointments” (P42, F, L)* and *“… it was getting to the point where every time I left the house I was like shit I’ve left something on, the house is going to burn down” (P307, F, D)*; and taking time off work or from childcare responsibilities.

Transportation was also cited as a barrier, especially for those in suburban areas of London and in Dorset. These were often exacerbated by mental or physical health problems: *“I don’t drive and with my anxieties I get nervous on public transport…” (P306, F, D).* Both mental health and screening professionals were aware that some service users may experience practical problems that can make attending screening harder for them, though no systematic approach to identify and overcome barriers for individual patients was evident: *“Think about negative symptoms of schizophrenia and chaotic lifestyle, in and out of hospital, drugs and alcohol, depression, all these things and plus I guess if they don’t go to the GP…” (P50, Psychiatrist, L).*

Service users found attending screening easier if the appointment was at a familiar location. Reminder letters and texts were said by some to be useful. Conversely, others found them intrusive: *“They kept inundating me with letters and that really made me paranoid” (P11, F, L)* and *“They wouldn’t leave me alone…they kept texting me and I’ve chosen not to have it and I’ve told my GP I don’t want to have it but they still send the letters…I just feel like they’re really trying to pressure you into it” (P112, F, D).*

## Discussion

Service users and health professionals have identified service delivery and client related factors that hinder or support uptake of different types of cancer screening. These factors were associated with five overarching themes: knowledge of screening programmes and processes, knowledge of and attitudes regarding mental illness, health service delivery factors, service users’ beliefs and concerns, practicalities for service users. Although common barriers and facilitators were identified for all screening types, our findings also indicated that there are barriers and facilitators specific to different types of screening. Common barriers and facilitators were also identified across participant characteristics, however interventions should be personalised to address individual differences. The identified barriers and facilitators in this study may also bear relevance to treatment adherence or care access if a problem is identified that requires treatment. Further research is required in this area to clarify this.

Our raw data identified similar concepts to those in the Theoretical Domains Framework [28] (see Fig. 1). The domains that appear to be key to service users’ cancer screening beliefs and behaviours are: knowledge; skills; social influences; memory, attention and decision processes; beliefs about consequences; motivation; emotion; behavioural regulation and environmental context and resources. The domains key to professionals’ promotion or conduct of cancer screening in people with mental illness are: knowledge; skills; social influences; professional role and identity; emotion; behavioural regulation and environmental context and resources.

### Strengths and limitations

This large qualitative public health study captures the views of both service users and health professionals regarding the barriers and facilitators to cancer screening uptake for all three of the UKs NHS screening programmes. Our methods and reporting meet COREQ (Consolidated criteria for reporting qualitative research) standards [30]. One strength of our study was that the interview schedule was based on a theoretical approach. The Theoretical Domains Framework was originally developed to study health practitioner behaviour change in implementation research but has been used also to explain health behaviour in general population samples [31, 32]. The domains include the main evidence-based factors influencing behaviour change, thus indicating the potential for development of theoretically based interventions to promote the desired behaviour by either service users or providers.

We did not include questions on the receipt of screening results in our interviews. It is likely that some of our identified barriers will relate to this too, for instance difficulties in receiving post when admitted to hospital. Additionally anticipation and receipt of results are likely to provoke anxiety in some service users. Service user participants included in the study reflected the target age range for the screening programmes and included men and women as well as representation from different ethnic groups. One limitation is the smaller number of men in the study. This could be linked to the well documented challenges in recruiting men to research [33, 34]. Additionally, there was a smaller sampling frame for this group. Furthermore only a small number of participants discussed bowel cancer screening. Although we found saturation for overall themes, saturation may not have been met for the male and bowel cancer screening subgroups. Most participants were asked how they would feel about bowel cancer screening, (in future if they were under 60), and participants generally discussed this type of screening less freely than other screening types suggesting a degree of embarrassment around this topic.

Participants were given the option to be interviewed face to face, at a range of locations, or over the telephone. This should mean our sample is inclusive of participants who may not have been able to attend if face to face interviews were offered exclusively. We also offered participants the choice of a male or female interviewer. Despite this, all participants chose a female interviewer or indicated they had no preference. Data analysis was primarily conducted by AC who has a psychology background and experience of working therapeutically with service users. This is likely to have influenced the interpretation of the data. To ensure a broad based approach to the subject the multidisciplinary team comprising a health psychologist, service user researcher, specialists in adult nursing, mental health nursing and primary care and public health sciences took part in each step of the data analysis process.

Screening professionals were primarily London based, though we used snowballing to recruit those working elsewhere. We obtained a good cross-section of types of staff working in mental health and screening services with wide variation in age and length of service. As with all studies where participants ‘opt in’, we acknowledge that both service users and professionals who place importance on pro-health behaviours may have been more likely to participate, as well as service users who have had particularly poor experiences. Despite this diverse views were evident.

Some research indicates that the type of mental illness, severity of illness or having untreated mental health issues can influence screening uptake [22, 35, 36]. The validity of grouping participants based on mental illness diagnoses has been questioned [11]. Individuals’ experience of mental illness varies between and within diagnoses and symptoms often overlap diagnoses [11]. Experience may also depend on whether the individual is receiving support from primary, secondary or inpatient services. We therefore decided not to define participants by their diagnosis but took an overarching, inclusive approach and, despite this, common themes were identified. This also ensures that our data can inform future interventions or service changes that will apply to diverse groups.

A novel finding of our study was that poor relationships exist between service users and health professionals involved in cancer screening. Some screening professionals also demonstrated less than positive attitudes to working with service users. It appears that our methods enabled participants to speak freely about these difficult topics.

### Comparison with other studies

Our findings concur with those of prior studies on cancer screening in service users [19–22] conducted elsewhere. These studies also found low income, lack of transport, embarrassment, fear of pain and of receiving a cancer diagnosis, adverse prior experiences, and lack of familiar care providers to be barriers. Ours is the first study to also consider bowel cancer screening in service users. Our work also confirms findings from the USA [23, 24], showing that the way services are organised, particularly where there is a lack of integrated care, can impact on cancer screening uptake in service users. In addition, our work suggests that some of the interventions found to be effective for increasing cancer screening uptake in the general population may not work for mental health service users. The evidence supports the use of postal [16] or short messaging service (SMS) reminders [37], but these may not be received or understood and may be found intrusive by service users. Similarly letters signed by a GP are useful in the general population, but may be off putting for some service users given reports of poor relationships with GPs. Addressing logistical barriers [16] will be relevant to service users. It is clear, however, that an individualised and mental health informed approach will be needed because barriers vary between individuals and some, such as difficulties leaving the house due to mental health symptoms, are specific to service users. Ours is the first study to combine the perspectives of three key stakeholders in the cancer screening process. This has enabled us to identify where views and experiences are at odds between groups and where they concur. For instance, cancer screening was felt to be important by all, but among some professionals there was disagreement as to who is responsible for promoting it in service users. This needs to be resolved before interventions to promote cancer screening uptake can be implemented.

### Implications for future policy and research

When deciding how to intervene, it should be noted that the identified barriers and facilitators to screening uptake are relevant at different stages of the screening process and, accordingly, different interventions will be needed for each stage. At the point of invitation, service users will not receive invitations or bowel cancer testing kits if they are admitted to hospital and will not be invited if they are not registered with a GP. Lack of integrated care means that mental health staff will not know if a service user is overdue for a test. When attending for screening, the range of barriers and facilitators for individual service users will vary, so a personalised approach to addressing and utilising these is needed. At the point of delivery of the screening test, again it is clear that individual needs will vary. Screening professionals are motivated to help, but often lack time or training to recognise and manage mental health needs. Though reactive measures are available, this places the onus on the service user to make a request for help, which may be difficult for some without formal systems for reasonable adjustments to care in place.

Future research should focus on interventions to promote informed uptake of cancer screening by service users that take account of the barriers and facilitators identified in this study and build on successful health promotion interventions in other disadvantaged populations. It should also be considered that interventions will need to be personalised to support individuals with their specific challenges. Tools could be developed to help, for instance a tool that combines the identification of necessary reasonable adjustments [38], the negotiation of logistical barriers [16] and a decision aid to ensure that choices are informed [39]. To overcome identified deficits in knowledge around screening and to reduce mental health related stigma, levels of professional and service user awareness should be established. Government could incentivise promotion of cancer screening, for instance through the ‘CQUIN’ system [40] that is currently used in UK secondary care mental health settings to promote cardiovascular care. Service providers could provide outreach where service users are contacted to discuss and assess their cancer screening needs; cancer screening liaison nurses have been successfully used for populations with intellectual disabilities [37] and health navigators have been found effective for addressing health disparities in African Caribbean communities [41]. Targeted clinics, akin to special care dentistry for people with physical or mental health conditions [42], could be developed. One London breast screening service already runs a dedicated clinic for women with special needs including mental illness [25]. Currently there is no systematic approach to promoting cancer screening uptake in service users. Evidence based approaches, such as those aforementioned, would address this and facilitate more equal access to services.

## Conclusions

Mental health service users face specific barriers to accessing cancer screening, thereby preventing equality of care. This study has identified facilitators that could be used to support the development of evidence based approaches to address this inequality. Systematic interventions that can be personalised should be developed and tested at the individual, policy and service-delivery level. It is essential that professionals from primary and secondary care such as GPs, nurses, psychiatrists and policy-makers work collaboratively to develop an integrated approach to addressing the needs of this population.

## Abbreviations

R&D, research and development; CMHT, Community Mental Health Team; NHS, National Health Service; GP, general practitioner; TDF, theoretical domains framework; SU, service user; SP, screening professional; MHP, mental health professional; P, participant; F, female; M, male; L, London; D, Dorset; A&E, accident and emergency; COREQ, Consolidated Criteria for Reporting Qualitative Research.

## Additional files

Additional file 1: Interview Schedules. Interview schedules for people living with a diagnosis of mental illness, for screening professionals and for mental health professionals. (DOCX 33 kb) Additional file 2: Tables S1 to S3. Barriers and Facilitators by participant and screening type. Themes, barriers and facilitators identified in the sample of people living with a diagnosis of mental illness, in the sample of screening professionals and in the sample of mental health professionals. (DOCX 19 kb) Additional file 3: Additional quotations from all participant groups. (DOCX 54 kb)
