# Supplementary material for: Influences on uptake of cancer screening in mental health service users: a qualitative study
Source: BMC Health Serv Res. 2016 Jul 12;16:257. doi: 10.1186/s12913-016-1505-4 (PMC4942968; doi:10.1186/s12913-016-1505-4)
Supplement: Additional file 1: — Interview Schedules. Interview schedules for people living with a diagnosis of mental illness, for screening professionals and for mental health professionals. (DOCX 33 kb) [file 12913_2016_1505_MOESM1_ESM.docx]

**Additional file 1a: Interview Schedule for Service Users**

**Introduction**

Thank you again for agreeing to take part in our study. We are looking at people’s views about bowel/smear/mammogram cancer screening (select as appropriate based on age and sex as discussed when confirming demographic information). We are interested in your views whether or not you have taken part in screening in the past. So in your case, my questions will be about mammogram/smear/bowel (as appropriate). I’m sorry if some of the questions sound a bit similar (omit if just one screening type applies).

I’m just going to begin recording our conversation now... (or not if participant is not happy for this)

Where more than one screening programme applies: which type of screening would you like to discuss?

***Knowledge***

- Can you tell me what you know about screening for mammogram/smear/bowel cancer? (prompts: why it is done, who is eligible, how often you are invited, who conducts the screening and what happens during screening)?
- Have you ever been invited to take part in (mammogram/smear/bowel) cancer screening? Can you tell me a little about that (when, how often)?
- For people who have experienced screening (and for each type of screening for which the participant is eligible): What was it like going for mammogram/smear screening (or doing the bowel cancer screening test)?
- What did you expect to happen when you went for screening?
- What was it like waiting for the results of your screen? (prompts: were you thinking about it much, did you feel anxious, did anyone help you to interpret the results, good/bad results?)
- For people who have not experienced screening (for each type of screening for which the participant is eligible):
- Can you say something about why you didn’t take up the offer of mammogram/smear/bowel screening? (choice, emotions, practicalities)?
- If you did go for screening, what would you expect to happen? Do you know what happens when people go for smear/mammogram cancer screening/do the bowel cancer screening test?

***Skills***

- Did/do you know what to do in order to obtain (bowel/smear/mammogram) screening if you wanted it? (prompts: who to ask, where to go, how to get there, what to do?)
- How able do/did you feel to make requests to suit your needs (e.g. for a longer appointment time, or to see a health professional of a particular gender)?
- How easy would it be for you to carry out the bowel cancer screening test (what help would you need, what is difficult, what would make it easier)?

***Social/professional role & identity***

- How did/do any beliefs or values about the way you live your life influence your decision whether or not to take up mammogram/smear/bowel screening? (e.g. cultural, religious, spiritual, family values)

***Beliefs about capabilities***

- How confident were you about going for mammogram/smear screening (and/or carrying out the bowel cancer test (collecting the samples for bowel cancer screening and sending them off)? (Did/(does lack of confidence affect whether or not you take part in screening?)
- What problems, if any, have you experienced going for screening?
- How did you overcome any problems (if at all)?
- How confident are you that you can take part in screening without feeling distressed before or during screening, or afterwards?
- Is there anything that would have made it easier for you?

***Beliefs about consequences (anticipated outcomes)***

- What do you think are the benefits to you of having cancer screening (if any)? (prompts: finding things earlier and getting them treated, reassurance that all is well)
- What do you think are the disadvantages to you of cancer screening (if any)? (prompts: that it might be a distressing procedure, involve unempathic health professional(s), fear that it could lead to relapse or exacerbation of mental health problems, beliefs about false positives and negatives, about further investigations that might be indicated, beliefs about cancer treatment, morbidity and mortality)

***Emotion***

- Did or do any emotional factors prevent you from taking part in screening, or make it harder for you? (prompts: such as feeling very anxious or depressed, not valuing yourself very much, or feeling embarrassed about your body, health worries, fears about the procedures involved, or being diagnosed with cancer)?

***Motivation***

- How important would you say cancer screening is to you?
- Are there other concerns that are more important? (e.g. balancing mental health needs – which is the most important, does it vary over time?)

***Memory, attention & decision processes***

- How do/did you make the decision to take part in mammogram/smear/bowel screening?
- Did or do you have any problem remembering to go for the screening appointment/collect the samples for bowel screening or posting them off?
- What did you think of the information that came with the invitation for screening? (e.g. Did you find the information easy to understand? Was anything unclear? How could they make things clearer?
- How much do you think that problems with your mental health influence whether or not you go for mammogram/smear/bowel screening? (e.g. in terms of deciding to go or remembering to go or take part)

***Social influences (norms)***

- Does/did anyone influence your decision to take part in screening? (who, how?)
- What do/did people close to you (partner, relatives, friends) think about you getting screened for mammogram/smear/bowel cancer? How important is/was it to take their views into account?
- How supportive are they – do they remind you/encourage you? (partner/relatives/friend) E.g. helping to understand the pros and cons, making phone calls to confirm or change appointments, reminding you to go, going with you and waiting in the waiting room, or being with you when you had the test?
- Do you think you have been influenced to go for screening by any media reporting eg on TV news or programmes? Or by any community events (groups they attend, local health promotion ads or literature)? Or by mental health or cancer charities or user-led organisations? Or by health professionals not directly doing the screening (e.g. care coordinator, psychiatrist, GP)?

***Environmental context & resources***

- How did you get to the screening appointment (for mammogram, smear only)? How would you get there (if not previously attended or if not intending to go)?
- How convenient is this? (e.g. transport difficulties; appointment times; how it fits in with work/ family; health/mobility problems)
- Are there any problems with the bowel screening procedure in terms of how and where this can be done?
- Do money issues make a difference to whether or not you take up screening?
- What about finding the time to fit it in...?
- Was there any time when you were invited but felt too ill to attend, or were in hospital for e.g. or felt too ill to make a decision about whether to have the screening or to be able to have/do the procedures?
- OTHER ACCESS FACTORS – e.g. does their address change frequently (might they have missed a postal invitation)?

***Behavioural regulation***

- Are there things you need to do in order to prepare to go for screening (e.g. to arrange to help them have their screening related needs met – longer appointments, choice of professional, use of medication to help you feel less anxious during screening, having Cognitive Behaviour Therapy or learning other coping or relaxation techniques to help with medical procedures, having discussions with screening staff in advance of your appointment)?
- What part have health professionals played in influencing whether or not you have screening? Have you had any discussion about it with e.g. GP, psychiatrist, nurse, therapist, care coordinator? What did you talk about? Was it helpful? Did they do anything? Is there anything you would have liked them to do?

***Finally...***

- Is there anything else you would like to say about what health professionals and health services might do to what might make it easier for people with mental health problems to decide whether or not to go for cancer screening?
- And what could be done to make the experience of cancer screening better for people with mental health problems?

**Additional file 1b: Interview Schedule for Mental Health Professionals**

**Introduction**

Thank you for agreeing to take part in our study. We are interested in exploring health professional views of cancer screening in people living with mental illness. By mental illness we mean people who have mental health problems which last for a year or more and have a serious impact on people’s lives. People living with mental illness may have diagnoses of schizophrenia, bipolar disorder, major depression, personality disorder or some anxiety disorders. We are looking at bowel, cervical and breast cancer screening for people living with mental illness*.*

I just want to ask you a few questions about your thoughts – please feel free to answer as honestly as possible. All information will be kept completely confidential. Are there any questions you would like to ask me before we start? (Turn on recorder)

***Knowledge***

- Can I start by asking what you know about the National Cancer Screening Programmes e.g. cancer types, who are eligible, frequency of screening?
- How do you see your role in terms of promoting cancer screening?
- What do you know about the link between severe mental illness and cancer survival?
- What factors do you think might contribute to fewer people living with mental illness getting screened for cervical and breast cancer? What about bowel cancer?

***Skills***

- Thinking about health promotion generally, what skills do you feel you have in this field?
- How easy is it to access the necessary information to help your patients with mental illness attend for screening (e.g. where, when, adjustments)?

***Social/professional role & identity***

- How far do you consider that it is part of your professional role to promote cancer screening in people with mental illness?

***Beliefs about capabilities***

- How easy is it to promote cancer screening to people with severe mental illness?
- How confident are you of overcoming any difficulties doing this if/when you encounter them?
- Would you say you experience any specific problems due to lack of confidence in this area?
- Is there anything that would make it easier for you?

***Beliefs about consequences (anticipated outcomes****)*

- What do you think are the costs and benefits of promoting cancer screening in this group? For example, what will happen if you do promote screening? Might there be disadvantages - heavier workload, dealing with anxiety/distress, causing relapse or exacerbation of existing mental illness?
- How do you feel if you omit to promote screening when you could?
- What do you think are the pros and cons for people living with mental illness regarding cancer screening?

***Motivation***

- How important would you say the promotion of cancer screening in this group is to you?
- Are there any financial or other incentives for you to engage people living with mental illness in cancer screening?
- Are there other concerns that take priority/are more important?
- Are there times when mental health needs outweigh cancer screening needs *(When? In which situations?)*

***Memory, attention & decision processes***

- How do you make the decision to promote screening or not? Is this something you usually do?
- How do you remember to promote cancer screening?
- How much of a priority is it (screening people living with mental illness)? Are there any protocols for care of this group of people?

***Environmental context & resources***

- Is there a system for promoting screening in your practice/workplace? For example is there someone who has overall responsibility? Are there computer prompts? Are there any physical health questionnaires you use with people living with mental illness which include cancer screening?
- Is there an impact on workload? In terms of staff resources, being able to offer sufficient time?
- Is there a system for re-inviting people who DNA?
- What happens for other groups, e.g. those with learning disabilities, physical disabilities?
- Could the procedure for these groups be applied to people living with mental illness?

***Social influences (norms)***

- Is there anyone at work who influences your decision to promote screening or not e.g. colleagues/managers? (who, how?)
- What is the general culture at work in relation to promoting cancer screening in this group?

***Behavioural regulation***

- Is there extra work or planning involved in organising screening for people living with mental illness? (longer appointments, choice of professional, use of medication, CBT, pre-visit discussions).

***Emotion***

- Do any emotional factors affect whether or not you promote or conduct cancer screening with people in this group? *(prompts: anxiety, concern, stress, burnout).*
- Finally, is there anything you would like to add to what we’ve discussed about cancer screening in people living with mental illness?

**Additional file 1c: Interview Schedule for Screening Professionals**

**Introduction**

Thank you for agreeing to take part in our study. We are interested in exploring health professional views of cancer screening in people living with mental illness. By severe mental illness we mean people who have mental health problems which last for a year or more and have a serious impact on people’s lives. People living with mental illness may have diagnoses of schizophrenia, bipolar disorder, major depression personality disorder or some anxiety disorders. We are looking at bowel, cervical and breast cancer screening for people living with mental illness*.*

I just want to ask you a few questions about your thoughts – please feel free to answer as honestly as possible. All information will be kept completely confidential. Are there any questions you would like to ask me before we start? (Turn on recorder)

***Knowledge***

- What is your experience of screening people living with mental illness?
- How do you think having a mental illness might affect the needs of this group when they undergo screening?
- What adjustments can you make to your practice to accommodate people living with mental illness? (is this a knowledge question?)
- What do you know about the link between severe mental illness and cancer survival?
- What factors do you think might contribute to fewer people living with mental illness getting screened for cervical and breast cancer? What about bowel cancer?

***Skills***

- Do you feel you have the necessary skills when it comes to screening people living with mental illness?
- What additional training, if any, do you think would be useful?

***Social/professional role & identity***

- Is it part of your professional role to promote cancer screening in people living with mental illness?

***Beliefs about capabilities***

- How do you promote screening in people living with mental illness? How easy is it to promote cancer screening in this group?
- How confident are you of overcoming any difficulties conducting screening in people living with mental illness?
- Would you say you experience any specific problems due to lack of confidence in this area?
- Is there anything that would make it easier for you?

***Beliefs about consequences (anticipated outcomes)***

- For those involved in promoting screening: What do you think are the costs and benefits of promoting cancer screening in this group? For example, what will happen if you do promote screening? Might there be disadvantages - heavier workload, dealing with anxiety/distress, causing relapse or exacerbation of existing mental illness?
- How do you feel if you omit to provide screening when you could?
- What are the pros and cons for people living with mental illness regarding cancer screening?

***Motivation***

- How important would you say cancer screening in this group is to you?
- Are there any financial or other incentives for you to engage people living with mental illness in cancer screening?
- Are there other concerns that take priority/are more important?
- Are there times when mental health needs outweigh cancer screening needs? *(When? In what situations?)*

***Memory, attention & decision processes (For those involved in promoting screening)***

- How do you make the decision to promote screening or not? Is this something you usually do?
- How do you remember to promote cancer screening?
- How much of a priority is it (screening people living with mental illness)? Are there any protocols for care of this group of people?

***Environmental context & resources***

- How does your system of screening work? *E.g do you provide opportunistic screening? Annual checks? Do you use computer prompts and reminders?*
- For people in breast cancer screening units/sexual health clinics: Do you know in advance if someone coming for screening may have mental health problems? If so, how do you prepare for this (if at all)?
- Is there an impact on workload? In terms of staff resources, being able to offer sufficient time?
- Is there a system for re-inviting people who DNA?
- What happens for other groups, e.g. those with learning disabilities, physical disabilities?
- Could the procedure for these groups be applied to people living with mental illness?

***Social influences (norms)***

- Is there anyone at work who influences your decision to screen or not e.g. colleagues/managers? (who, how?)
- What is the general culture at work in relation to screening for cancer in this group?

***Behavioural regulation***

- Are there any procedures to encourage screening in this group of people? E.g longer appointments, choice of professional, use of medication, CBT, pre-visit discussions)?

***Emotion***

- Do any emotional factors affect whether or not you conduct cancer screening with people in this group? *(prompts: anxiety, concern, stress, burnout).*
- Finally, is there anything you would like to add to what we’ve discussed about cancer screening in people living with mental illness?
