# Supplementary material for: Influences on uptake of cancer screening in mental health service users: a qualitative study
Source: BMC Health Serv Res. 2016 Jul 12;16:257. doi: 10.1186/s12913-016-1505-4 (PMC4942968; doi:10.1186/s12913-016-1505-4)
Supplement: Additional file 2: Tables S1 to S3. — Barriers and Facilitators by participant and screening type. Themes, barriers and facilitators identified in the sample of people living with a diagnosis of mental illness, in the sample of screening professionals and in the sample of mental health professionals. (DOCX 19 kb) [file 12913_2016_1505_MOESM2_ESM.docx]

**Table S1.** Themes, barriers and facilitators identified in the sample of people living with a diagnosis of mental illness

| **Theme** *(Theoretical Domain)* | **Barrier to uptake** | **Cervical** | **Breast** | **Bowel** | **Facilitator to uptake** | **Cervical** | **Breast** | **Bowel** |
| --- | --- | --- | --- | --- | --- | --- | --- | --- |
| **Knowledge of screening** *(Knowledge; skills; social influences; memory, attention & decision processes, beliefs about consequences)* | Not knowing what to expect or what to do | ✓ | ✓ | ✓ | Wanting to be informed | ✓ | ✓ | ✓ |
|  | Unsure of need for screening | ✓ | ✓ | ✓ | Understanding of benefits of screening | ✓ | ✓ | ✓ |
|  | Difficult to process information | ✓ | ✓ | ✓ | Encouragement | ✓ | ✓ | ✓ |
| **Motivation to attend**  *(Motivation)* | Additional burden | ✓ | ✓ | ✓ | Feeling ‘health conscious’ | ✓ | ✓ | ✓ |
|  | Mental health symptoms reduce motivation for self care | ✓ | ✓ | ✓ | Being anxious to avoid further health problems | ✓ | ✓ | ✓ |
|  |  |  |  |  | Physical symptoms (e.g. finding a lump) | ✓ | ✓ | ✓ |
| **Anticipation of negative or positive experience**  *(Emotion)* | Past negative experience | ✓ | ✓ | ✓ | Past positive experience | ✓ | ✓ | ✓ |
|  | Embarrassment | ✓ | ✓ | ✓ |  |  |  |  |
|  | Traumatising | ✓ | ✓ | ✓ |  |  |  |  |
|  | Fear of bad news | ✓ | ✓ | ✓ |  |  |  |  |
| **Accommodation of mental health needs by NHS staff and services**  *(Behavioural regulation)* | Lack of understanding of mental illness in screening professionals | ✓ | ✓ | X | Staff being understanding | ✓ | ✓ | X |
|  | Screening environment aggravates mental health symptoms | ✓ | ✓ | X | Staff knowledge of mental illness | ✓ | ✓ | X |
|  | Staff can be rushed | ✓ | ✓ | X |  |  |  |  |
|  | Staff can be rough | ✓ | ✓ | X |  |  |  |  |
|  | Exclusion from GP registers | ✓ | ✓ | ✓ |  |  |  |  |
| **Access to screening**  *(Environmental context & resources)* | Appointment booking | ✓ | X | X | Familiar location | ✓ | ✓ | ✓ |
|  | Transport difficulties | ✓ | ✓ | X | Reminders | ✓ | ✓ | ✓ |
|  | Difficulty remembering appointments | ✓ | ✓ | ✓ |  |  |  |  |
|  | Difficulty leaving the house due to mental health problems | ✓ | ✓ | X |  |  |  |  |
|  | Taking time off | ✓ | ✓ | X |  |  |  |  |
| **Relationships with health care staff in general**  *(Emotion)* | Made to feel like a burden on health service | ✓ | ✓ | ✓ | Good relationship with GP | ✓ | ✓ | ✓ |
|  | Poor relationship with GP | ✓ | ✓ | ✓ | Good relationship with Practice Nurse | ✓ | ✓ | ✓ |
|  | Diagnostic overshadowing | ✓ | ✓ | ✓ | Continuity of care | ✓ | ✓ | ✓ |
|  | Stigma of mental illness | ✓ | ✓ | ✓ |  |  |  |  |

**Table S2.** Themes, barriers and facilitators identified in the screening professional sample

| **Theme** *(Theoretical Domain)* | **Barrier to uptake/delivery** | **Cervical** | **Breast** | **Bowel** | **Facilitator to uptake/delivery** | **Cervical** | **Breast** | **Bowel** |
| --- | --- | --- | --- | --- | --- | --- | --- | --- |
| **Approaches to meeting complex needs**  *(Knowledge; skills, environmental context & resources)* | Lack of knowledge of severe mental illness | ✓ | ✓ | X | Understanding of emotional and practical barriers to screening uptake for PLWDMI | ✓ | ✓ | ✓ |
|  | Lack of time | ✓ | ✓ | X |  |  |  |  |
| **Attitude to PLWDMI**  *(Emotion; professional role & identity)* | Find complex patients difficult | ✓ | ✓ | X | Staff motivated to encourage screening for all groups | ✓ | X | X |
| **Communication skills**  *(Skills)* | Communication skills training not available to all | X | ✓ | X | Importance of good communication skills recognised | ✓ | ✓ | X |
|  |  |  |  |  | Confidence to screen anyone associated with good communication skills | ✓ | ✓ | X |
| **Integrated care**  *(Environmental context & resources; behavioural regulation)* | No means of knowing patient needs in advance | ✓ | ✓ | ✓ | Practice nurses can access patient record | ✓ | ✓ | ✓ |
|  | Computer systems not linked | ✓ | ✓ | ✓ | Reactive measures in place if notice given | ✓ | ✓ | X |

**Table S3.** Themes, barriers and facilitators identified in the mental health professional sample

| **Theme** *(Theoretical Domain)* | **Barrier to uptake/promotion** | **Cervical** | **Breast** | **Bowel** | **Facilitator to uptake/promotion** | **Cervical** | **Breast** | **Bowel** |
| --- | --- | --- | --- | --- | --- | --- | --- | --- |
| **Knowledge and confidence to promote screening**  *(Knowledge; skills; social influences)* | Lack of knowledge of programme and /or procedures | ✓ | ✓ | ✓ | Health promotion seen as their role | ✓ | ✓ | ✓ |
|  | Promotion of screening not prioritised | ✓ | ✓ | ✓ | Aware that PLWDMI are at risk of cancer | ✓ | ✓ |  |
|  | Lack of a structured behaviour change approach | ✓ | ✓ | ✓ | Diagnostic overshadowing known to be a problem | ✓ | ✓ | ✓ |
| **Integrated care**  *(Skills; Environmental context & resources; behavioural regulation; emotion)* | Lack of collaboration between healthcare services | ✓ | ✓ | ✓ | Understanding emotional and practical barriers to screening uptake for PLWDMI | ✓ | ✓ | ✓ |
|  | Lack of physical health expertise | ✓ | ✓ | ✓ |  |  |  |  |
|  | Stigma of mental illness | ✓ | ✓ | ✓ |  |  |  |  |
| **Health service delivery factors**  *(Environmental context & resources; behavioural regulation; professional role & identity)* | Cancer screening promotion not their responsibility | ✓ | ✓ | ✓ | Willingness to promote screening | ✓ | ✓ | ✓ |
|  | Patient’s mental state | ✓ | ✓ | ✓ | Cancer screening promotion included in routine health promotion | ✓ | ✓ | ✓ |
|  | Lack of resources | ✓ | ✓ | ✓ |  |  |  |  |
