# Supplementary material for: Influences on uptake of cancer screening in mental health service users: a qualitative study
Source: BMC Health Serv Res. 2016 Jul 12;16:257. doi: 10.1186/s12913-016-1505-4 (PMC4942968; doi:10.1186/s12913-016-1505-4)
Supplement: Additional file 3: — Additional quotations from all participant groups. (DOCX 54 kb) [file 12913_2016_1505_MOESM3_ESM.docx]

**Additional file 3: Online Supplementary Quotes**

**Key**

Participant= P

Service User=SU

Screening Professionals= SP

Mental Health Professionals=MHP

Male=M

Female=F

**Mental Health Service User/ Service User (SU)**

**Theme: Knowledge of screening**

Barrier: Not knowing what to expect or what to do

*“I didn’t know what to expect...I thought that was what they were going to do put me through a tunnel” (P 4, SU, F)*

*“For about the last couple years I’ve been getting letters from them saying about cancer screening and they send a kit, but I don’t know if I really understand a hundred percent how to work this kit” (P 47, SU, M)*

Barrier: Unsure of need for screening

*“As in like OK let’s talk about why you need it and making me feel that I need to have it rather than feeling like well if they are only bothered to send a letter and nobody wants to talk about it then it makes you feel that well obviously I don’t really need it” (P 110, SU, F)*

*“There isn’t anything … unless you find symptoms yourself like breast screening or other cancers or cancer of the bowel you don’t automatically get screened for them” (P 7, SU, F)*

*“Well that’s the reason because I don’t feel anything at the moment so I thought if I need to do it then I will” (P 61, SU, M)*

Barrier: Difficult to process information

*“I’ve just come out of a depression type of thing; I’m not really out of it properly, so at those sort of times I lose my confidence. I can’t do anything; little things can become very, very big. The fact that I am feeling a little better in myself I think maybe I might be able to read and follow the instructions” (P 47, SU, M)*

*“I’ve got to read a lot of things. I find it hard to concentrate” (P 22, SU, F)*

Facilitator: Wanting to be informed

*“Give them more information about it, leaflets, booklets for them to read up on it, send them to social people who have done it, network” (P 4, SU, F)*

*“If they had a Facebook page telling you information about what cervical screening is, what it involves, is it painful, is it not painful because a lot of people my age don’t even know what it is” (P 112, SU, F)*

*“I think it might help if, before it came, if they told me, particularly the bowel one, that it will be coming through the post, that I will be getting this little kit. I think the Doctor should tell their patients” (P 41, SU, F)*

Facilitator: Understanding of benefits of screening

*“I know for a fact that the earlier you find out about these things the more chance that the success in beating or fighting these things” (P 47, SU, M)*

*“It’s better to find out if you have got it if you found out sooner than later. The sooner you find out the more chances it can be cured” (P 32, SU, F)*

*“They can identify the abnormal or pre-cancerous cells to prevent cancer from developing” (P 306, SU, F)*

Facilitator: Encouragement

*“I know in the past that if I keep putting off going to the doctor, keep putting it off and keep putting it off and my friends then have to encourage me book an appointment” (P 15, SU, F)*

*“I swing between like I said worrying thinking well if I have got cancer I don’t care and then the next day I can be really high and really worried because I wished something on myself and then panic and go and get it. But a lot of the time it’s my friend and my mum who will push me and say you should get this sorted out, it’s probably nothing but just in case” (P 307, SU, F)*

*“Every time I went to my GP (General Practitioner) and he brought it up I’d say can we do it next time, can we do it next time. He did give me that leeway because he knew it was me and not another one of his patients and so he waited until the time was right for me…he reminded me and said that it’s time it needs to be done and I said to him, I explained my fears and said I really, really hate having it done, I really don’t want to have it done. He’s going I know I understand but it’s really important and he is very soothing, he’s a very good GP” (P 44, SU, F)*

**Theme: Motivation to attend**

Barrier: Additional burden

*“A letter is just a letter and I’ve had so many letters recently it’s just been washed away with all the other letters and at the moment it’s the least of my priorities so whether it’s highlighting a priority of it or, yes highlighting the priority of it to me it’s the least of my priorities” (P 110, SU, F)*

*“I’ve only had one smear in my life so I’m overdue, I think it was about 3 or 4 years ago I had my last one. I haven’t been because I’ve just had so much going on in life recently you know you put it off” (P 307, SU, F)*

*“Once you are over 50 most women rightfully in all rights should receive mammograms or breast screening and normal people just get on with it and don’t worry about it really and I think they think well the mentally ill should just get over it and get on with things and not complain or find things so hard but I think what they forget is that the mentally ill population some of them do find everyday life quite difficult” (P 11, SU, F)*

Barrier: Mental health symptoms reduce motivation for self-care

*“Sometimes when you are feeling low you don’t tend to look after yourself” (P 15, SU, F)*

*“It just seems like nothing is really worth it anyway....it doesn’t matter if you were to have it (cancer) because it would do everyone a favour” (P 23, SU, F)*

*“If I were to develop breast cancer or bowel cancer then that for me would be, well that would be the end of my life because my bi-polar is so severe and has been for so long that I would probably be quite relieved if I did develop a form of cancer that was fatal” (P 111, SU, F)*

Facilitator: Feeling ‘health conscious’

*“I think it’s really important and I think that there should be a lot of awareness raising” (P 39, SU, F)*

*“I tend to think about my health quite holistically so I just try to stay healthy in all respects” (P 39, SU, F)*

*“When it comes to cancer and all that I am quite conscious because I have got quite a few people in the family” (P 32, SU, F)*

Facilitator: Being anxious to avoid further health problems

*“Because you’ve already got that mental illness, whatever it could be, and then if you have to deal with breast cancer or cervix cancer or any type of cancer, it’s more treatment, more drugs, more doctors, more admissions and another disease that you will have to deal with” (P 32, SU, F)*

*“Well I just think if I have all these problems then I don’t want added problems, you know” (P 10, SU, F)*

Facilitator: Physical symptoms

*“I’ve always said I’ll probably die of ignorance because I put things off until I’ve got symptoms and then it’s like oh I’d better get this sorted out now” (P 307, SU, F)*

*“It was only when I felt this big lump that I thought oh shit I’d better do something about it” (P 307, SU, F)*

*“If I find my body starts malfunctioning in certain ways I would hope that I would sort of say well alright is any of these feelings I’m getting is it any of those things I’ve spoken to other people about, this cancer that cancer and then I may as I say go to my GP and you know” (P 47, SU, M)*

**Theme: Anticipation of negative or positive experience**

Barrier: Past negative experience

*“It was all very peculiar and there was a lot of nurses hovering around and I didn’t feel very comfortable. Not only that but somebody opened the door when they were there and it was a man so he poked his head around the door. I wanted to complain…” (P 109, SU, F)*

*“It [cervical cytology] was done initially by the nurse at the GP surgery and it was excruciatingly painful. I think that does put me off the whole business of having the screening… the whole experience was quite difficult and frightening and quite painful” (P 39, SU, F)*

*“I would say that’s predominantly what puts me off [the unpleasantness of procedure] and I remember feeling as though my breasts had been cut off, I felt very embarrassed to have to go in the waiting room with other people just while they were sort of getting some paperwork together, you weren’t even sent anywhere quiet to sit” (P 35, SU, F)*

Barrier: Embarrassment

*“I’ve got nobody to discuss it with for a start, I know I can discuss it with my partner but I’m talking about a male, I didn’t have anybody to talk to I was too embarrassed” (P 114, SU, M)*

*“It was pretty horrific because you have to lay there, put your legs open and I think that’s why I haven’t been back really” (P 115, SU, F)*

*“Very uncomfortable and embarrassing and a little bit unpleasant and a little bit painful” (P 11, SU, F)*

Barrier: Traumatising (experience of screening)

*“Trauma shut me down to the point I couldn’t communicate anything” (P 35, SU, F)*

*“It just makes you feel, I don’t know as if you’ve been raped. That’s how it feels...You are assaulted” (P 15, SU, F)*

*“There was one smear though that happened that that was a trigger to me remembering some childhood abuse afterwards… But I coped with it and it wasn’t debilitating if you know what I mean, I got over it OK” (P 109, SU, F)*

Barrier: Fear of bad news

*“Well I’m frightened I suppose, don’t want to know, what I don’t know I don’t worry about so...” (P 114, SU, M)*

*“The last test I had, it had abnormal cells and I was meant to go back but I didn’t…I don’t really want to know” (P 115, SU, F)*

*“I think I was scared…It’s like burying my head in the sand” (P 20, SU, F)*

Facilitator: Past positive experience

*“The clinic … where I went they were amazing there, they were so good and I think as well it helped that they were geared up, the beds were the right type of beds and I found a smear there 100 times better than anywhere else … the conversation was appropriate while it was happening, the supervised member they were very comforting in a way and they also explained every single thing that they were going to do and I think that makes you automatically relax which makes it less painful” (P 309, SU, F)*

*“I went to an STI clinic as well which was just so much better, I just didn’t feel anything it was just perfect… everyone is just extremely open and it’s the most judgeless place you can go into and very down to business and matter of fact and it’s just what you need… for me now and going forward in the future I would happily go to a sexual health clinic to have it done” (P 44, SU, F)*

**Theme: Accommodation of mental health needs by National Health Service (NHS) staff and services**

Barrier: Lack of understanding of mental illness in screening professionals

*“Last time I went, the lady was laughing because I was shaking but I thought because I told her I was on medication that causes my hands to tremor...she should have been more understanding” (P 3, SU, F)*

*“When I’ve had smear tests in the past I’ve been upset and I’ve cried and they think it’s because I’m in pain and it’s actually because I’m just a bit distressed and feel uncomfortable in that situation and sometimes you can get a bit of a battleaxe nurse who will say come on now it’s not that bad and that’s not very helpful, not very understanding” (P 306, SU, F)*

Barrier: Screening environment aggravates mental health symptoms

*“I’m a voice hearer, there are times when you can talk out to the voices so it would be difficult if you were by yourself and you just couldn’t control that” (P 23, SU, F)*

*“If I’m at a very low place I may have paranoia so waiting in a room full of other people so paranoia and social anxiety so that’s not good, that really isn’t good when you are in that place” (P 22, SU, F)*

*“It’s the waiting around that I can’t stand. I get very impatient and very anxious. Quite a few times I’ve wanted to just leave because I’m waiting for so long and I can’t tolerate it” (P 55, SU, F)*

Barrier: Staff can be rushed

*“She didn’t seem to know what she was doing; it was like she was rushed” (P 19, SU, F)*

*“I feel whenever I go and see my GP I feel as if they are trying to push you out before you can sit down and really talk to them” (P 15, SU, F)*

*“I’ve never had a rushed appointment in my life compared to the mammogram and it’s such a personal thing in such a sensitive painful area it’s really left me with quite significant trauma …I’m not happy the way the screening is done I just think, also I remember them being terribly rushed so it was like, as soon as you went in you could see they were anxious and in a panic mood and concentrating on the paper and not even looking at you” (P 35, SU, F)*

Barrier: Staff can be rough

*“I didn’t realise they pull you about so much…I don’t mind going but it’s a bit traumatic you know they’re pulling, pulling, tugging away at you” (P 10, SU, F)*

*“I went to run away the lady couldn’t do it so she called another colleague in and this lady did the smear but she was actually brutal I felt but maybe in that moment she was brutal but having managed to get the cells she may have saved me a second visit” (P 35, SU, F)*

Barrier: Exclusion from GP registers (London only)

*“He (the GP) told me to leave the local area because I wasn’t liked around there” (P 11, SU, F)*

*“I asked for my disability living allowance, physical needs not mental and I said to the GP can you write down my issues and he only wrote about mental and I was very disappointed so I complained…because of my medication it causes aggression as one of the side effects so then I blasted them didn’t I” (P 3, SU, F)*

*“I used to go the surgery and say could you see me and they would say we are closing now but somebody after me they’d see so I kicked up such a fuss that in the end they wanted me to go but I thought they did me a favour because I get more quality service where I am now” (P 3, SU, F)*

Facilitator: Staff being understanding

*“When you have the actual procedure there’s a big difference from someone just being very medical about something in comparison to somebody who is being thoughtful and respecting you and that you are in a situation where it’s a bit awkward at times, embarrassing or uncomfortable and it’s nice to have a bit of reassurance and being told what’s going to happen is, what they are doing next, is really important” (P 309, SU, F)*

*"He’s (GP) so accommodating, if I say I really can’t get down he will come out or he will send someone out” (P 55, SU, F)*

*“I think if they are nice and quite relaxed talking to you it puts you at ease a lot whereas if you’ve got someone quite militant you think it’s not very nice” (P 104, SU, F)*

Facilitator: Staff knowledge of mental illness

*“Maybe even to give the people doing the smears some kind of training on how to be more sensitive to people who do suffer with mental illnesses who might be more anxious about the procedure than other people” (P 306, SU, F)*

*“There could be some training with regard to maybe bedside manner that when you get a distressed patient. Maybe she (the GP) was worried that I was going to get aggressive, I wasn’t I was just getting very, very distressed, maybe that’s why she got fearful and spiky and almost. Her body language changed completely she actually got up and made herself bigger and made herself a bigger presence in the room and then I don’t think she knew what she was doing” (P 42, SU, F)*

*“I mean if it’s a person who is going to take that and it’s going to be useful for you, interaction in a positive way then it is important but if that’s going to make the interaction more difficult then it’s going to be not helpful” (P 31, SU, F)*

**Theme: Access to screening**

Barrier: Appointment booking

*“In my old surgery it would just be like ‘no we don’t have any appointments at all.’ Even if you’ve just been in to see the doctor and said he wants to see you tomorrow, they’ll be like ‘well we don’t have any appointments. I’m afraid you’ll have to phone in the morning’… usually with the kids being up and getting off to school it would be really difficult to get that 8.30am appointment” (P 115, SU, F)*

*“If you want to see the GP you have to specify and they ask you why, you know I can’t understand this you know I go to see the GP to get it booked and they ask you why, there might be others there and you have to talk about this” (P 47, SU, M)*

*“When I’m booking an appointment sometimes they can be a bit brusque with me” (P 48, SU, F)*

Barrier: Transport difficulties

*“I don’t drive and with my anxieties I get nervous on public transport and also some days when I’m feeling low and depressed I’m quite tired and it’s quite a walk to the bus stop and then it just seems like such a lot of effort. Obviously I’m not working at the moment and taxis are expensive and my partner can’t always drive me around” (P 306, SU, F)*

*“Well just making the effort and getting there initially because I did have panic attacks so as I said making the effort in getting there, especially if I had to go on transport” (P 305, SU, F)*

*“Parking is quite difficult at the hospital because it’s quite confusing where to go” (P 108, SU, F)*

Barrier: Difficulty remembering appointments

*“If I don’t put anything in my phone diary I forget so, I forget what I did yesterday so” (P 104, SU, F)*

*“I put everything in my diary and important things have alarms on them as well. I’ve learnt over the years that depression messes with your memory and it has such an impact on everything that unless I write it down I can’t be sure that I said it or that I did it or that I’m going to do it” (P 44, SU, F)*

Barrier: Difficulty leaving the house due to mental health problems

*“There have been times when I’ve been very low to the point whereby I don’t even want to go out the house and I’m regularly missing appointments and I can’t deal with face to face contact” (P 42, SU, F)*

*“When I’ve been particularly bad and very low I struggle to leave my home so whatever official appointments or non-official appointments I have set up they just go to the wayside” (P 44, SU, F)*

*“If I’m having a bad day, because some days I struggle to leave the house, for example today I’m having a good day so I’m able to get out and about but on a bad day it can be really difficult to get out the door” (P 306, SU, F)*

Barrier: Taking time off

*“I don’t like having to take time off work and also I suppose telling people at work you don’t want to have to tell M managers what you are doing” (P 39, SU, F)*

*“usually I have to make arrangements, childcare arrangements or arrangements for my son to be dropped off at school so that I can get down to the hospital or get to the GP” (P 39, SU, F)*

Barrier: Reminders

*“I don’t think a reminder would make me no I think, well I think if the doctor said or if I had a problem or the doctor said about it I’d probably take more notice of it” (P 114, SU, M)*

*“They kept inundating me with letters and that really made me paranoid” (P 11, SU, F)*

*“They wouldn’t leave me alone…they kept texting me and I’ve chosen not to have it and I’ve told my GP I don’t want to have it but they still send the letters, they’ve probably sent about 5 letters and I just feel like they’re really trying to pressure you into it” (P 112, SU, F)*

Facilitator: Familiar location

*“That sort of thing makes a difference where you go if it’s a familiar place and if it’s a person that you know” (P 31, SU, F)*

*“That is my hospital, that is, where I had all my children, all my medical reports are there” (P 4, SU, F)*

*“The second time I had it done it was quite close to where I work in one of the buildings I think linked to [the hospital] but yes so it was more convenient and I could pop down there while I was at work. Those kind of factors I think make a difference, convenience, fitting round work” (P 39, SU, F)*

Facilitator: Reminders

*“I think having a reminder makes it a lot simpler than having to think oh where’s my calendar to go write it down because you can always have something going on and you forget” (P 3, SU, F)*

**Theme: Relationships with health care staff in general**

Barrier: Made to feel like a burden on health service

*“I don’t darken his door any more than I have to [GP] …I feel that I’m taking up his time, I’m wasting time so I only go if I have to go because it’s time for a medication review” (P 24, SU, F)*

*“I thought that they weren’t actually testing the results properly because I had mental illness or because they didn’t like me or my family or whatever in the area where I’ve been living in in … but they just given me false results and maybe I did have cancer but they weren’t telling me the full truth or weren’t putting me in the picture about my own health. I just seemed to be another burden on the NHS” (P 11, SU, F)*

Barrier: Poor relationship with General Practitioner (GP)

*“I went into the doctors and said look I’m in a really bad way I think I’m going to kill myself, I’ve got everything I need to do it, and he just said oh try not to do it I’ll phone you on Tuesday – this was like a week away – and see how you are. So I just went and did it the next night” (P 115, SU, F)*

*“I very rarely went to the doctors because I just felt as if I had been judged...You feel very exposed for a cervical smear especially when you feel as if you are being judged” (P 23, SU, F)*

*“I feel that he’s not really listening (GP) ...when I do book an appointment and they brush me off I just don’t want to go back” (P 15, SU, F)*

*“I’ve seen the doctor many times but no one has ever mentioned the fact that I’ve received these letters (cervical cancer screening reminders) and I still haven’t had it done so it’s obviously just a computer system that automatically sends it out” (P 110, SU, F)*

Barrier: Diagnostic overshadowing

*“I think that’s the problem- I’m not taken seriously” (P 3, SU, F)*

*“When I said I had a lump he thought that I was imagining it or gave me the impression that he thought I was imagining it” (P 23, SU, F)*

*“As soon as the doctor found out that I had mental health problems he said oh all the symptoms you are experiencing are anxiety and they said get in touch with your MHP, maybe you should go back and get some counselling and I was completely dismissed. He refused to see me and then a week later I had to go to A&E because I’d actually got a water infection that had spread up to my kidneys and that’s why I was feeling so unwell because I’d got a fever with it. So it wasn’t in my head” (P 306, SU, F)*

Barrier: Stigma of mental illness

*“You feel like you are not important and you don’t matter as much as someone without mental health problems” (P 306, SU, F)*

*“I got a referral to a gynaecologist again and on the referral letter at the bottom it had all these mental health related things on the bottom of the letter and it was all about suicide and self-harm and emotional and single personality, and I was like oh my God this person is going think I’m really difficult to deal with and that’s going to make him not very nice to me” (P 31, SU, F)*

*“It’s not surprising that we die younger because we are not liked” (P 11, SU, F)*

*“I keep my mental health issues quite quiet to be honest because I don’t want people judging me because oh she’s got schizoaffective disorder, she’s a nutter” (P 112, SU, F)*

Facilitator: Good relationship with General Practitioner

*“I think having a good GP surgery like for example they are very sympathetic” (P 3, SU, F)*

*“I think you also build that relationship with, especially with somebody like a GP who you need to trust, it’s not just a mechanical interaction it’s building up a relationship” (P 19, SU, F)*

Facilitator: Good relationship with Practice Nurse

*“It’s all down to the nurse and how the nurse is with you…Be friendly and open and you want to know that the nurse does this all the time and for her it’s not weird because I think for one the other end it’s rather weird, it’s a weird thing to do. So unusual, uncomfortable so just to know that the nurse does this all the time and for her it’s just run of the mill day to day activity is helpful and for her to develop some sort of rapport with you - discuss the weather, or politics or whatever” (P 19, SU, F)*

*“When you have the actual procedure there’s a big difference from someone just being very medical about something in comparison to somebody who is being thoughtful and respecting you and that you are in a situation where it’s a bit awkward at times, embarrassing or uncomfortable and it’s nice to have a bit of reassurance and being told what’s going to happen is, what they are doing next, is really important” (P 309, SU, F)*

*“The nurse that did it was really good, she explained everything before she did it and again it was the reassurance that if it hurts I’ll stop but it won’t because it’s really quick” (P 307, SU, F)*

Facilitator: Continuity of care

*“Continuity would be helpful” (P 19, SU, F)*

*“I know it’s not possible to always have the same people but I think a little bit of familiarity knowing what you are going to expect is a big pull in itself” (P 35, SU, F)*

*“I mean for me there’s a nurse at the GP …she’s really nice and I’ve known her for a long time and she’s the sort of person who makes, and I’ve been there before when I’ve hurt myself and stuff and she’s been really nice about that and she hasn’t been nasty, she’s been sympathetic and helped me sort things out so I think she’s a nice person so an experience like that is better with someone who you know already and who is sensitive” (P 31, SU, F)*

*“Emotionally I mean I always go back to the same nurse at the GP” (P 39, SU, F)*

Screening Professionals (SP)

**Theme: Approaches to meeting complex needs**

Barrier: Lack of knowledge of mental illness

*“I think it’s easy to get confused between people who have got mental illness and people who have got mental disability” (P 59, SP (Primary Care))*

*“No we’ve never done any training on women that could possibly come with mental illness, I think it’s something that you are expected to pick up on the job” (P 56 SP (Mammography))*

*“Obviously again when something like that happens in clinic the radiographer doesn’t know what the condition is, they might know that the lady is upset and they might know that she has got a carer but they don’t, and again they’re not MHPs so they don’t know and understand whether this is a permanent condition, is it something that with medication is treatable so all we can do it capture the information that is at the time but obviously appreciate that sometimes when the lady comes back the next time it maybe a completely different scenario” (P 54 managerial staff (breast cancer screening unit))*

Barrier: Lack of time

*“On a high pressure work day if you’ve got a very busy clinic it can just be very hard for the clients because we can’t give them as much time as we want or that they might necessarily need because we’ve got to see X amount of people and you don’t want to keep other people in the waiting room waiting particularly with some of our satellite units as well like the little vans that we use there’s only a finite amount of space” (P 57, SP (Mammography))*

*“Generally speaking when I do screening I do know the history of the person, how much regard I’ve taken that into account or how much I’ve really taken that on board would depend on how much of a rush I’m in because it’s not obviously normally overtly relevant to the fact that somebody needs to have a cervical screen whether or not they’ve got mental illness” (P 59, SP (Primary Care))*

*“Sometimes it’s an explanation, sometimes it’s them just wanting to stop, sometimes they just want to stop in between, they don’t want to talk, some say ‘it’s very painful, one minute I need to sit down’ and you just think oh, obviously me being anxious knowing that I’ve got another patient in 6 minutes I try and do it as fast as I can” (P 56, SP (Mammography))*

*“With any patient you might not have time to deal with anything other than what the consultation is about…” (P 60, SP (Primary Care))*

Facilitator: Understanding of emotional and practical barriers to screening uptake for service users

*“A long wait here, the waiting room is chaotic and it’s not a particularly nice environment and you have to be fairly organised to come to a clinic like this to get a ticket and so the access isn’t really that easy for people” (P 13, SP (Sexual health))*

*“She had a problem being undressed in front of a stranger which is obviously quite difficult when you are doing a mammogram so I asked how I could help her with that. She said she wasn’t sure we’d just have to get on with it, she did want to attend the screening, she did realise the reasons why and I suggested to her would you like me to turn the lights off and she went yes actually I would. So we did a mammogram with no lights on, in a completely dark room which was a little bit strange but we got on with it” (P 57, SP (Mammography))*

*“If someone finds it upsetting again you would get a feel of the person and make sure, well I’d want to make sure I was sensitive to that and I would think is it so upsetting that it’s going to be worse for her to have this smear and what are her risks” (P 51, SP (Primary Care))*

*“In my role of reminding them that they are overdue to have this done I think the only thing I would do differently is that I would, as I would with any patient, try to tailor my advice to their own needs. In that sense it might take me longer to explain to someone who had those difficulties why this was important and why it should be a priority for them... So I think it is important that we recognise that some people need more information, reassurance, advice and general input in order to adhere to the programme” (P 60, SP (Primary Care))*

*“I think we have to respond to the fact that people with severe mental illness have different needs in terms of their information needs and the way we communicate all the issues. We have to respond to that not by allowing the issue to be forgotten about or deprioritised but by making sure that they get what they need which may be longer appointments or more frequent appointments or other sources of material, information or material” (P 60, SP (Primary Care))*

**Theme: Attitude towards service users**

Barrier: Find complex patients difficult

*“Sometimes it could just be them being very difficult from the start, being sometimes even aggressive or rude… some women are quite the opposite they seem very needy and they need to sit down and can I have some more time and it’s just a mammogram obviously to me” (P 56, SP (Mammography))*

*“If you’ve got five or six reminders it’s unlikely you are going to cover all of them in 10 minutes particularly as none of them are actually the reason why this consultation was arranged” (P 60, SP (Primary Care))*

Facilitator: Staff motivated to encourage screening for all groups

*“It should be something that is promoted because certainly cervical screening has a financial incentive for general practice so it is in their interest to encourage as many women on their list to have cervical screening” (P 2, SP (Sexual health))*

*“I think it is really important for us to promote breast screening to the wider population especially for ladies who might not be able to be self-aware of the different issues that go on with the breasts” (P 58, SP (Mammography))*

**Theme: Communication skills**

Barrier: Communication skills training not available to all

*“I think communication course is very important… we’re not all just radiographers some of my colleagues are assistant practitioners and the radiographers are supported in ‘OK we’ll let you go on eventually to the communication course’ but APs are not given that right even though they do screen these women and they may get a women who could be mentally unwell” (P 56, SP (Mammography))*

*“If they are stressed and tense it’s very difficult to get them to cooperate because the machine we have is quite awkward, there is a lot of leaning and lifting of arms and small movements so it’s a stress, sometimes they are not listening and it’s difficult to convey what you want them to do” (P 58, SP (Mammography))*

*Facilitator: Importance of good communication skills recognised*

*“I think once again just giving them time and allowing them to tell you what their concerns are. Giving them that opportunity not just bulldozing them in to something and once again reiterating they don’t have to have this done, if it’s making them feel uncomfortable they’ve always got the opportunity to say stop either during the procedure or if they want to just not go ahead with the procedure then that’s fine” (P 57, SP (Mammography))*

*“I think being able to listen to what they want so how they are feeling (is important)” (P 58, SP (Mammography))*

*“It’s very important with everyone but it’s obviously probably that little bit more important to be really clear with people about the procedure, full information and support with any questions or queries and a very, very important thing with any intimate examination that the individual feels in control” (P 2, SP (Sexual health))*

Facilitator: Confidence to screen anyone is associated with good communication skills

*“I feel quite confident that I’ve met a lot of different people, different personalities and it helps you to adapt in different situations” (P 58, SP (Mammography))*

**Theme: Integrated care**

Barrier: No means of knowing patient needs in advance

*“There is no way of knowing, so sometimes you are just presented by a situation that you were not prepared for at all…If you don’t know about any mental disability then you haven’t accommodated have you, you assume she’s well, just because you can’t see it physically you are assuming she’s well” (P 56, SP (Mammography))*

*“So we don’t get very much information from the GP’s practice it’s pretty much the client’s name, address, phone number and anything else that we feel we need we’ll ask the client there and then but that’s really all we get from a GP’s practice...they wouldn’t tell us anything about the patient’s mental health and they wouldn’t tell us about any other medical condition that they would have which is sometimes confusing for patients and clients because they say but you know all of this and we’re like well no we don’t, we don’t actually really get a great deal of information” (P 57, SP (Mammography))*

Barrier: Computer systems not linked across healthcare settings

*“If they get the letter from the NHS agency they may not know that they have a mental illness so it makes it harder because they don’t always have access to records” (P 51, SP (Primary Care))*

*“We get informed if somebody hasn’t turned up for one but that would be as a letter that would end up in their notes rather than a screen reminder message which is linked to the actual system” (P 60, SP (Primary Care))*

*“In a GP practice they’re in a slightly better position because they’ve got the information so they can check on the records and say well actually you had it two weeks ago or we’ve got a result from three months ago because our records are completely confidential to any other service, this is the sexual health service we can’t do that so if we can’t actually find that information out maybe we have to make a judgement to go ahead and do it” (P 2, SP (Sexual Health))*

Facilitator: Practice Nurses can access patient record

*“How they can help is when they get a DNA notification from us to flag that up on their system then the next time they get access to the woman whether it be because she comes in for an appointment or pops into see the nurse to then maybe say ‘did you receive your appointment?’ and then it opens up the channel about why they haven’t attended. So it’s actually using the people that have got the access” (P 54, SP (Mammography))*

*“So there is something that primary care can do to alert people to their screening programme, and if they don’t attend all of these notes will be flagged, they will all be identified or the notes will be flagged so that opportunistically when the people come in for their medication or for their prescription that it’s been asked you didn’t attend your mammogram, your screening for breast cancer, I wonder why not” (P 16, SP (Public Health))*

Facilitator: Reactive measures in place if advance notice of patient need is given

*“I think sometimes telling us prior to the examination might be helpful because then we can be prepared, for example if you’ve got somebody with quite a severe physical difficulty it’s helpful to have two radiographers in the x-ray room. If you have somebody who might have violent or aggressive tendencies it’s safer to have two radiographers in there… Also to give them more time as we mentioned five minutes is tiny so if you know that you can accommodate them by giving them a longer appointment then it’s a lot easier” (P 57, SP (Mammography))*

*“The practice would be very understanding if I gave a double appointment to someone because they know that I wouldn’t do that without good reason and I’d want other nurses to do the same. But they may not know in advance that they’d need a double appointment so that perhaps would be good if they did” (P 60, SP (Primary Care))*

*“Well definitely hopefully give them more time, be prepared myself in the sense that OK this is the next lady I need to make sure, you just prepare yourself mentally as well for us I need to be a bit more patient, I need to get the room ready, explain things. It’s almost like you are able to prepare yourself to be at your best as well as the clinician, maybe prepare the paper to hand it to them, maybe if you need to show them drawings. You will be able to deal with it I think better if you were expecting something” (P 56, SP (Mammography))*

*“So I think people with severe mental illness certainly … the consultation may be longer, you have to be able to have the time which I know can be a problem sometimes in GP practices you are quite limited with time, the service I use we don’t have a time limit so … we could see someone for 45 minutes if that person needed that time” (P 2, SP (Sexual health))*

Mental Health Professionals (MHP)

**Theme: Knowledge and confidence to promote screening**

Barrier: Lack of knowledge of screening programme and/or procedures

*“One of our questions we were asking people which is on RIO [NHS electronic patient record system] is something about, I think it’s towards the men… have you gone for your cancer checks for bowels or whatever and we have no idea what age you are meant to do that, it’s not on there at all… so it’s clarity really as to what screening is available for people so that we do know what to be promoting” (P 308, MHP)*

*“One of the outcomes for me is I think I need to look at the details of cancer screening programmes so that I’m really clear so that I can support how often people should go for what and how and when and what are the indicators and how would they do that and where would they access that” (P 38, MHP)*

*“If they raise a symptom then definitely they would be prioritised to be investigated …if they haven’t mentioned it then no one would know” (P 37, MHP)*

*“It’s not easy at the moment to promote it because we don’t, as clinicians, we don’t necessarily know where we have to go to get the information to promote it” (P 302, MHP)*

Barrier: Promotion of cancer screening not prioritised

*“Our priority is, you know, engagement, stabilisation and not cancer screening” (P 17, MHP)*

*“Cancer risk is rife amongst the population anyway and there is quite an awareness about it anyway but with this group it’s so easy to overlook that because there are so many other more apparent problems that they come into contact with us with so it wouldn’t always be at the forefront of our mind” (P 46, MHP)*

*“The sad truth is that people tend to be more concerned about the medication that they are obliged to take for their mental health issues than they are about lifestyle, diet, things like that” (P 21, MHP)*

*“We talk about the relationship with GP, ongoing physical health problems, any previous diagnosed conditions, medication for physical health, when they last saw the dentist, nutrition screen, optician things like that, lifestyle issues and obviously we work alongside other services to look at sexually transmitted diseases and things like that but in terms of cancer screening I’d say that my knowledge is quite limited” (P 40, MHP)*

*“I think it (promotion of cancer screening) varies from person to person, some people maybe aren’t as keen and don’t think it is that important” (P 101, MHP)*

*“As a team it had been hit and miss especially since multidisciplinary and some of the team members are social workers and they would say well we don’t know what to look for or what questions to ask or they weren’t comfortable with doing patients weight and stuff like that” (P 308, MHP)*

Barrier: Lack of a structured behaviour change approach

*“They all talk about their physical health and stuff like that so sometimes, like I said, I do find myself talking about health issues, what they can do and how they’re feeling; period pains, headaches, all sorts” (P 30, MHP)*

*“My job as an OT (Occupational Therapist) is looking at people’s routines and their lifestyle and if they are drug taking and unhealthily eating and all of those things then I would definitely talk to them about all of those things and what they’re doing with their time. Just basically all areas of their life really but it’s not really like in a formal way it’s more woven into just normal conversation” (P 37, MHP)*

*“I think also because we’ve done so much for these patients as well maybe we’ve deskilled them in some way for taking responsibility for their own healthcare needs as well so they wait for us to do it for them” (P 308, MHP)*

Facilitator: Health promotion seen as their role

*“Our remit is to consider someone’s physical health as much as their mental health but our remit I guess is also about promoting positive engagement with health services which that could be part of” (P 18, MHP)*

*“You are always thinking about someone’s physical health care because of the medications that they are on but you are also thinking about their physical health care because clearly that is going to affect their mental health so it is important” (P 50, MHP)*

*“Sometimes it’s as simple as literally getting them there on time and to the right place so it can be a very practical thing. I think sometimes it can be a language thing they just need a bit of extra support to understand... it might be just they want somebody else with them so that they are clear about what has been discussed or agreed by the doctor” (P 43, MHP)*

Facilitator: Aware that service users are at risk of cancer

*“We know that the mortality rates of being with psychosis is 20 years less thereabouts than people without … that’s appalling really and it is COPD and cancers and diabetes that are the causes of that high mortality rather than schizophrenia, people don’t die from schizophrenia they die from lung cancer, breast cancer, cervical cancer, airways disease, heart attacks, things like that” (P 38, MHP)*

*“Cancer is roundabout the third leading cause of death within the population, it not only causes death it causes disability, suffering, reduced quality of life and angst for carers and loved ones and relatives as well as the individual involved so there’s a real priority in looking at early identification intervention and particularly within people with severe mental illnesses who for a range of factors are at a greatly increased risk” (P 5, MHP)*

*“I would imagine the patients we look after anyway have a lifespan that is 15, maybe 20 years sometimes less than the average person and sometimes that is due to medication and I imagine they probably don’t go to as many physical health checks up as maybe other people do” (P 103, MHP)*

Facilitator: Diagnostic overshadowing known to be a problem

*“If there is primary diagnosis of depression or psychosis that gets sort of, I think GP’s get influenced by that and if somebody starts coming in saying I’ve got physical health problems they think oh it’s caused by the psychosis or that’s caused by the depression” (P 28, MHP)*

*“Part of it may be to do with GP’s having difficulty perhaps understanding or interpreting someone’s presentation and not recognising a physical health concern or not focusing on physical health concern and wanting to focus on mental health concern when it isn’t what the person has come for” (P 17, MHP)*

*“I think part of it may be to do with GP’s having difficulty perhaps understanding or interpreting someone’s presentation and not recognising a physical health concern or not focusing on physical health concern and wanting to focus on mental health concern when it isn’t what the person has come for. So it might be that the GP, yes doesn’t strike a balance I suppose between the two.” (P 18, MHP)*

*“I got the impression from the literature that perhaps there’s maybe an assumption that mental health patients are often sort of they think oh that’s probably their mental health but I don’t think they do do that on the ward that I work on. They will examine things even if they appear to be psychological in origin” (P 37, MHP)*

**Theme: Integrated care**

Barrier: Lack of collaboration between healthcare services

*“Often I find that a lot of the feedback between GPs and secondary care is very one way…the only way you’ll get feedback from the GP is if you really chase them up” (P 43, MHP)*

*“Sometimes I’ll ring a GP and they’ll say there is no one available and they’ll call me back and it will be 7pm at night…It would just be so much easier if there was more of a centralised system whereby we could get access to more information” (P 50 MHP)*

*“As a nurse or a social work say they are having to liaise with the GPs sometimes it’s harder for them to get their points across or their concerns across” (P 308, MHP)*

*“I think that’s probably more difficult for us sometimes because we use different computer programmes … sometimes knowing when all our patients have appointments can be a bit difficult for us unless we get a letter or they come with a letter sometimes we don’t always know what they are due” (P 103, MHP)*

Barrier: Lack of physical health expertise

*“I don’t want to over generalise but I think specialist mental health settings the majority of staff are focused specifically on mental health issues and in general terms their training reflects this” (P 5, MHP)*

*“Generally I think people’s MHPs’ confidence in addressing physical health issues is probably quite low unless you have had particular experiences that have led you to develop confidence ... it feels a bit beyond our comfort zone” (P 18, MHP)*

*“I know that people in mental health services we’re really pushing to try and get people to join up the physical and mental health side of things and I think often we tend to focus just on our specialism at the expense of the physical health in general” (P 21, MHP)*

Barrier: Stigma of mental illness

*“There’s the general attitude towards mental illness in primary care… for example, forensic histories and mental, quite severe and enduring anti-social histories are best avoided” (P 17, MHP)*

*“Somebody with a mental health diagnosis was saying they went to their GP because they had a chest infection and the receptionist said to them when they were checking them in said ‘oh you people are always coming to the GP, you are always taking up all of our time, and you spend hours in there’” (P 18, MHP)*

*“Some GPs are also quite harsh, when I was a CPN [mental health nurse] myself I had quite a few clients who got struck off by the GP because either they are perceived as not following the rules or they are perceived as rude to the receptionists” (P 8, MHP)*

*“Sometimes they’ve had bad experiences with GPs. They find the GPs don’t understand about mental health particularly well so they feel that the GP has just dismissed them whenever they try and complain about a physical problem” (P 28, MHP)*

Facilitator: Understanding of emotional and practical barriers to screening uptake for service users

*“I suppose attending these appointments, sometimes reading ... the appointment date and time and what it’s for, obviously their mental state as well and yes money, and their motivation or actually not realising how important it actually is, not having the knowledge” (P 40, MHP)*

*“Think about negative symptoms of schizophrenia and chaotic lifestyle in and out of hospital, drugs and alcohol, depression, all these things and plus I guess if they don’t go to the GP and they see us and we’re not very good at it because we don’t really know much about it” (P 50, MHP)*

*“I think it’s for some who don’t particularly trust health professionals or don’t particularly trust health services then ... they may be quite dismissive of or quite reluctant to engage with processing that kind of information” (P 18, MHP)*

*“Our patients complain about waiting times to see a GP. A lot of them are quite anxious sat in a waiting room” (P 17, MHP)*

**Theme: Health service delivery factors**

Barrier: Cancer screening promotion not their responsibility

*“This is something I would have thought their GP would have or maybe assertive outreach somebody who works more long term with the patients” (P 106, MHP)*

*“There is a balance to be struck between what is part of your role because it’s for the benefit of the service user and what is, there is a bit of a danger that if you as a mental health professional take on a lot of the work that otherwise might be done by GP or a primary care service then it’s slightly, it takes away the responsibility from the primary care service” (P 18, MHP)*

*“I suppose there’s that kind of assumption whose role and responsibility it is maybe? Is this something that the GP should be advocating, is it something that the GP should be monitoring or is it that this should be everyone’s responsibility and maybe there’s obviously we’ve got KPIs, we’ve got key performance indicators that nurses have to and I suppose healthcare professionals have to meet and sometimes when it’s not on a screening it won’t be asked” (P 40, MHP)*

Barrier: Patients’ mental state

*“I think if somebody is suffering from some of the anxiety syndromes, severe anxiety again you’d need to be mindful of how any additional stress would affect them physically and you would have to balance that against the benefits of actually trying to persuade them to go through the screening process” (P 302, MHP)*

*“I think we’ve got to get this in the right area there is no point somebody like me doing it in PICU it’s got to come from home treatment teams and CPN [Community Psychiatric Nurse]. The areas where unless you actually say to them you have to you write it on a care plan and they have to book it because that’s actually the only way you’ll get it done” (P 1, MHP)*

*“This is an acute treatment place where patients are generally here for 72 hours, some are long term but very rarely. We deal and specify straight within the mental health and it’s such a quick turnover if they did have, if we started the role of screening we wouldn’t be there to follow it through” (P 106, MHP)*

*“I think if someone is acutely paranoid, acutely ill, very paranoid, very disturbed that’s not the time to approach them around screening” (P 38, MHP)*

Barrier: Lack of resources

*“It’s finding the opportunity and having the time” (P 1, MHP)*

*“I suppose cost wise then they are going to be taking less resources out of the system later on but the problem is later on is many years on and so it’s very hard then to get those costings and prove how much money you are saving because it’s not everybody is just so interested with the here and the now aren’t they, not what you are going to save in 10 years or 15/20 years time” (P 308, MHP)*

*“How we mobilise, where does that resource come from” (P 38, MHP)*

Facilitator: Willingness to promote screening

*“I think we should be promoting screening for cancer definitely it’s just we maybe need a bit more education about when and who we should be promoting it for” (P 103, MHP)*

*“I mean I haven’t had anybody coming in to speak to us or have even questioned us this is the first time ever so I think it’s good, what you are doing is good…we are becoming more aware of what we should be doing really” (P 29, MHP)*

*“I deliberately didn’t look anything up in anticipation of this interview but actually one of the outcomes for me is I think I need to look at the details of cancer screening programmes so that I’m really clear so that I can support how often people should go for what and how and when and what are the indicators and how would they do that and where would they access that” (P 38, MHP)*

Facilitator: Cancer screening promotion included in routine health promotion

*“With promoting cancer screening it would really be something that we would be looking at when we get all the initial assessment information together and promoting annual physical reviews with GPs so because we know that there tends to be a problem with people with enduring mental health problems keeping their annual physical review appointments that’s something that we would help to facilitate and provide support and discussion on” (P 304, MHP)*

*“We pulled stuff from RIO, stuff from the Rethink physical health check and stuff to tie in with the CQUIN requirements we have so it all fits in to about a three page health assessment” (P 105, MHP)*

*“This is the one we should do which is the physical health check devised by Rethink and a few professionals that we actually know and I used to work with, so everyone in our Trust who is on CPA will have that physical health check that includes blood pressure, weight, all the calculation of the BMI, give some basic information for example if you are a woman the CPN should talk to about you need to arrange for a Pap explain a little bit why you need a Pap etc.etc” (P 8, MHP)*
